# Enhanced transcriptome-wide RNA G-quadruplex sequencing for low RNA input samples with rG4-seq 2.0

**DOI:** 10.1186/s12915-022-01448-3

**Published:** 2022-11-13

**Authors:** Jieyu Zhao, Eugene Yui-Ching Chow, Pui Yan Yeung, Qiangfeng Cliff Zhang, Ting-Fung Chan, Chun Kit Kwok

**Affiliations:** 1 Department of Chemistry, and State Key Laboratory of Marine Pollution, City University of Hong Kong, Kowloon Tong, Hong Kong, SAR China; 2 School of Life Sciences, and State Key Laboratory of Agrobiotechnology, Chinese University of Hong Kong, Shatin, Hong Kong, SAR China; 3 MOE Key Laboratory of Bioinformatics, Center for Synthetic and Systems Biology, School of Life Sciences, Tsinghua University, Beijing, 100084 China; 4 Beijing Advanced Innovation Center for Structural Biology & Frontier Research Center for Biological Structure, School of Life Sciences, Tsinghua University, Beijing, 100084 China; 5 Tsinghua-Peking Center for Life Sciences, Beijing, 100084 China; 6 Shenzhen Research Institute of City University of Hong Kong, Shenzhen, 518057 China

**Keywords:** rG4-seq 2.0, G-quadruplex, Transcriptome, dU adapter cleavage, cDNA library preparation

## Abstract

**Background:**

RNA G-quadruplexes (rG4s) are non-canonical structural motifs that have diverse functional and regulatory roles, for instance in transcription termination, alternative splicing, mRNA localization and stabilization, and translational process. We recently developed the RNA G-quadruplex structure sequencing (rG4-seq) technique and described rG4s in both eukaryotic and prokaryotic transcriptomes. However, rG4-seq suffers from a complicated gel purification step and limited PCR product yield, thus requiring a high amount of RNA input, which limits its applicability in more physiologically or clinically relevant studies often characterized by the limited availability of biological material and low RNA abundance. Here, we redesign and enhance the workflow of rG4-seq to address this issue.

**Results:**

We developed rG4-seq 2.0 by introducing a new ssDNA adapter containing deoxyuridine during library preparation to enhance library quality with no gel purification step, less PCR amplification cycles and higher yield of PCR products. We demonstrate that rG4-seq 2.0 produces high-quality cDNA libraries that support reliable and reproducible rG4 identification at varying RNA inputs, including RNA mounts as low as 10 ng. rG4-seq 2.0 also improved the rG4-seq calling outcome and nucleotide bias in rG4 detection persistent in rG4-seq 1.0. We further provide in vitro mapping of rG4 in the HEK293T cell line, and recommendations for assessing RNA input and sequencing depth for individual rG4 studies based on transcript abundance.

**Conclusions:**

rG4-seq 2.0 can improve the identification and study of rG4s in low abundance transcripts, and our findings can provide insights to optimize cDNA library preparation in other related methods.

**Supplementary Information:**

The online version contains supplementary material available at 10.1186/s12915-022-01448-3.

## Background

RNA structure plays an important role in biological function and regulation including but not limited to transcriptional and translational processes, RNA splicing, polyadenylation, mRNA localization and stabilization [1–6]. During the last decade, many methods were developed to study RNA structures by combining enzymatic and chemical probing with the next-generation sequencing (NGS) technique, yielding comprehensive in vitro and in vivo structural maps of RNA on a transcriptome-wide level, and providing new insights into RNA biology [7–16]. One unique RNA secondary structure, RNA G-quadruplex, which is composed of G-quartets connected by loop nucleotides, is of emerging importance in both chemistry and biology fields and has been associated with diseases such as cancers and neurological disorders [6, 17–21]. In 2016, we introduced RNA G-quadruplex sequencing (rG4-seq) [22] for the identification of in vitro rG4 on a transcriptome-wide scale that can facilitate further exploration and study of in vivo rG4 structure and function. Recently, we have refined a few steps of the rG4-seq experimental procedures [23] and also developed a bioinformatics pipeline known as rG4-seeker [24]. To date, rG4-seq has been successfully applied to study the rG4s in human [22], plants [25], bacteria [26], and plasmodium [27].

**Fig. 1.**
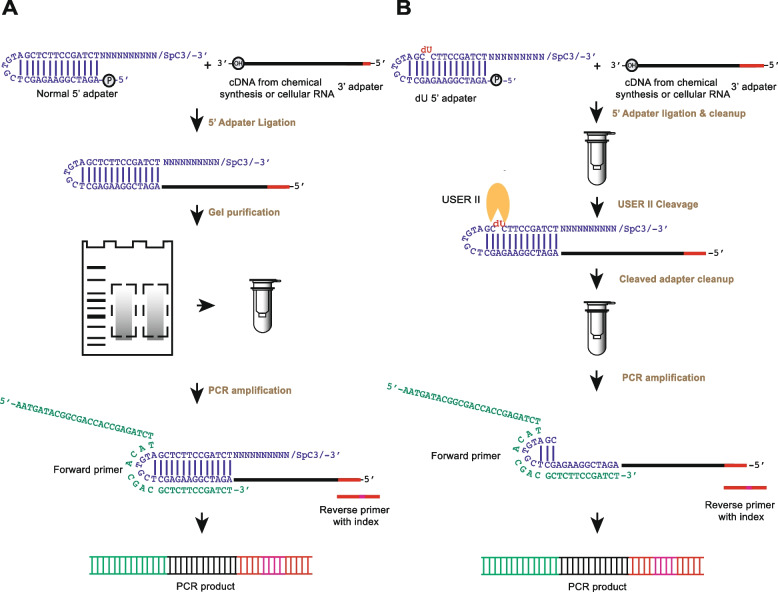
Comparison of the experimental strategy of rG4-seq and rG4-seq 2.0 from 5‘ adapter ligation to PCR amplification. The sequences of the oligonucleotides used can be found in Additional file 1: Table S1. The experimental pipelines that use **A** normal 5′ adapter and **B** 5′ dU adapter are shown (blue). In **A**, the normal 5′ adapter ligates to the cDNA (black) with 3′ adapter sequence (red) followed by gel purification before performing PCR amplification. The 6nt index region in the reverse PCR primer is in pink. In **B**, dU adapter is used for the ligation, and after the column purification, the enzymatic cleavage step is performed with cleavage enzyme (USER II, orange pacman) followed by column clean-up before the PCR amplification. USER II enzyme cleaves the 5′ adapter at the dU position as indicated in red in the 5′ dU adapter. The dU adapter contains the same secondary structure as the normal 5′ adapter and carries a 5′ phosphate at its 5′ end to ligate to incoming cDNA with a 3′hydroxyl, and a C3 spacer group at its 3′ end to block and avoid self-oligomerization. The 10 random nucleotides (N10) serve as a template for the hybridization of any incoming cDNA

It is noted that the 5′ adapter ligation step is one of the critical steps in rG4-seq and several other cDNA library preparation methods [22, 28–30]. Previously, we invented a hairpin DNA adapter to hybridize with the incoming cDNA and perform the ssDNA ligation mediated by T4 DNA ligase [31]. Over the years, this identical or highly similar strategy has been widely applied in the ligation step of new library preparation methods [23, 32–36]. The major limitation of such hairpin DNA adapter is that the secondary structure of the adapter may interfere with the base-pairing of forward primer with the cDNA template. Furthermore, the complicated gel purification step after the 5′ adapter ligation also lengthens experimental processing time and increases the samples loss. Both factors result in lower PCR amplification efficiency, requiring more PCR cycles (or input RNA) to obtain sufficient double-stranded DNA (dsDNA) for sequencing (Fig. 1A).

In this study, we have introduced a new 5′ DNA adapter that contains a deoxyuridine (5′ dU adapter) and removed the gel purification step (Fig. 1B) in the library preparation of rG4-seq, which we referred as rG4-seq 2.0. Using model oligonucleotides, we successfully applied rG4-seq 2.0 to varying polyA-RNA inputs (500 ng, 100 ng, 30 ng, and 10 ng) and showed that the data are of high quality, reproducibility and accuracy for transcriptome mapping and rG4 calling. Compared to the old method [23], rG4-seq 2.0 yielded higher PCR products with fewer PCR cycles and improved rG4 calling outcome by mitigating a significant nucleotide bias in rG4-induced RTS site identification persistent in the old method. We reported for the first time the in vitro rG4 map of HEK293T cells, and discussed the relationships among RNA amount inputs, read coverage and transcript abundance for rG4 mapping. Furthermore, we propose a standard recommendation for better judgment of RNA amount inputs and sequencing depth for specific rG4 studies based on the target abundance in the transcriptome.

## Results and discussion

### USER II cleavage

#### Effect of reaction time on normal and 5′ dU adapter cleavage by USER II

5′ adapter ligation is an essential step before PCR amplification in the cDNA library preparation of rG4-seq. We hypothesize that the 5′ adapter with strong secondary structure may affect the downstream processes such as PCR amplification efficiency (Fig. 1). To test this, based on our previously developed normal 5′ adapter (Fig. 1A), we first designed a modified 44-nt dU adapter with a dU nucleotide at the 24th nucleotide position from 5′ end to replace the T nucleotide (Fig. 1B). To process the dU adapter, Thermolabile USER II enzyme (USER II) was used, which creates the abasic site and further cleaves out the phosphodiester backbone to produce 2 DNA fragments, with the size of 23 nt and 21 nt, from the 5′ dU adapter. To explore the appropriate reaction time for dU cleavage, we tested and compared the cleavage rate with different reaction time on both dU and normal adapters.

**Fig. 2.**
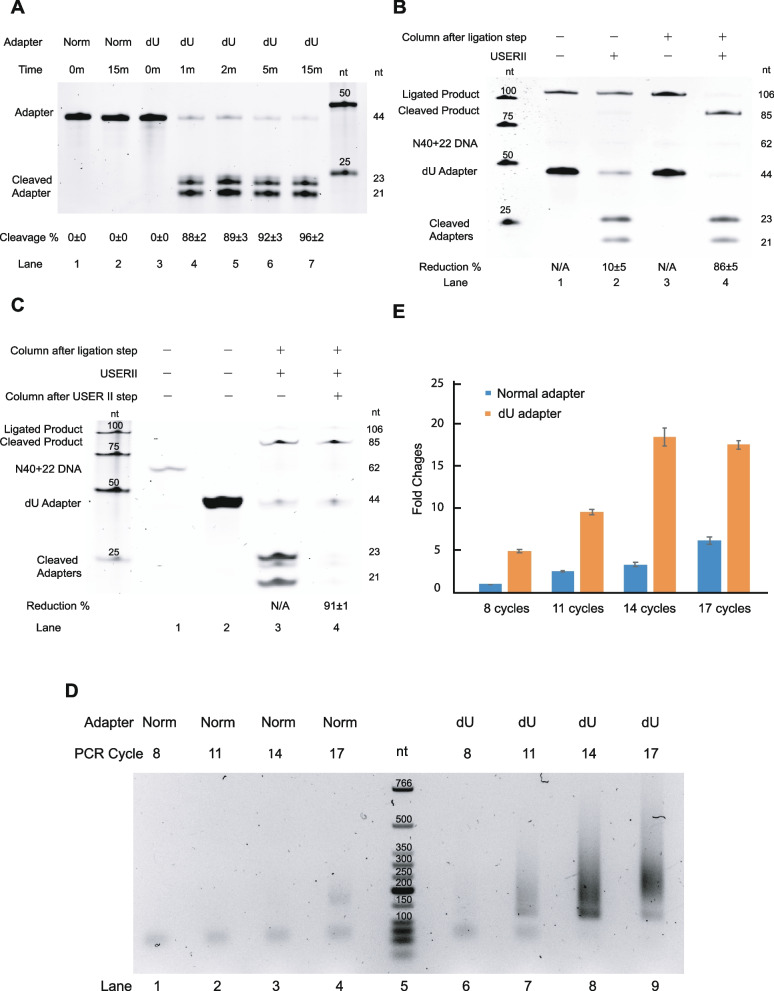
Optimization of experimental procedures. **A** The effect of reaction time on the normal and 5′ dU adapter cleavage. The figure shows the cleavage of both normal (lane 1–2) and dU adapter (lane 3–7) with USER II at different reaction time. **B** The effect of column purification on the USER II cleavage after the ligation step. The 106-nt ligated product bands without USER II cleavage before and after column purification (lanes 1 and 3) are defined as a reference for calculation. Equation 3 is used for calculating the reduction rate of ligated product (see the “Methods” section). **C** The effect of column purification on cleaved adapters removal after USER II treatment. The cleaved adapters generated from the USER II treatment (lane 3) are used as the reference to calculate the reduction rate of the cleaved adapters after column purification followed by the USER II step (lane 4). The N40+22 DNA only (62 nt in lane 1) and dU adapter only (44 nt in lane 2) are used as size references. Equation 5 is used for calculating the reduction rate of cleaved adapters (see the “Methods” section). **D**, **E** The effect of normal adapter and dU adapter on the PCR amplification. Agarose gel image of the PCR result is shown in panel **E**. PCR products of the library generated with 8, 11, 13 and 17 PCR cycles using a normal adapter (Norm) and 5′ dU adapter. Comparison of the relative quantity of PCR product is shown in panel **F**. 8 cycles of PCR product with normal adapter is defined as 1. The DNA size marker indicates the fragment size. The errors showed are standard deviation. nt=nucleotide. Three biological replicates are performed in **A**–**C** and two biological replicates in **D**–**E**. Raw data values are provided in the Additional file 8

We performed the USER II treatment at different time points (0, 1, 2, 5, 15 min) on dU adapter and 0 and 15 min for the normal adapter as negative controls, and ran them on denaturing PAGE gel to determine if the 5′ dU adapter (44 nt) was cleaved into shorter fragments (23 nt and 21 nt) by USER II. Compared to the normal 5′ adapter (Fig. 2A, lanes 1–2), the dU adapter showed varying degrees of cleavage with increasing incubation time (Fig. 2A, lanes 3–7). The cleavage rate reached over 96% in 15 min reaction time (Fig. 2A, lane 7), while there was no cleavage observed at 0 min for dU adapter (Fig. 2A, lane 3). As expected, no cleavage was observed on the normal adapter in both 0 min and 15 min (Fig. 2A, lanes 1 and 2). We also tested dU cleavage with longer time including 30, 60 and 120 min, and found there was no significant difference on the cleavage efficiency compared to 15 min (Additional file 1: Fig. S1A). Therefore, we found that USER II could specifically cleave dU adapter at the dU position with a highest cleavage rate in 15 min of reaction time.

#### Effect of reaction buffer on normal adapter and 5′ dU adapter cleavage

In the workflow (Fig. 1), the 5′ dU adapter cleavage step is performed after the 5′ adapter ligation in the cDNA library preparation, e.g. in our rG4-seq method [23]. In order to study whether the cleavage efficiency of USER II enzyme is compatible with normal reaction conditions used in the 5′ adapter ligation step, we have assessed two different factors including reaction buffer and PEG 6000 concentration (Additional file 1: Fig. S1B).

We first compared the cleavage efficiency of USER II enzyme in both CutSmart (CS) (recommended by vendor) and quick ligation (QL) buffer (also used in 5′ adapter ligation). No cleavage was observed regardless reaction buffer type without USER II addition (Additional file 1: Fig. S1B, lanes 1 and 2), while 97% cleavage rates were obtained after USER II treatment for both CS and QL buffer (Additional file 1: Fig. S1B, lanes 3 and 4), suggesting that the cleavage efficiency of USER II enzyme was not affected by reaction buffer. Although the commercial QL buffer includes 7.5% PEG 6000 originally, QL buffer got a cleavage rate as high as CS buffer (Additional file 1: Fig. S1B, lanes 3 and 4). We next tested the effect of different PEG 6000 concentration with QL buffer on the dU adapter reduction rate. Our result showed that from 7.5 to 17.5% PEG 6000 concentration, the adapter cleavage rates were all around 97–98% (Additional file 1: Fig. S1B, lanes 4–6), which were highly similar to the one under CS buffer (Additional file 1: Fig. S1B, lane 3).

In short, we showed that USER II could work normally with QL buffer and different concentrations of PEG 6000 condition.

#### Effect of USER II concentration on 5′ dU adapter cleavage

Besides reaction time and buffer conditions, we have also investigated the effect of USER II concentrations ranging from 0.05 to 0.2U/μl on the cleavage efficiency. Five minutes of reaction time was chosen in this test as 5′ dU adapter could be mostly cleaved by the USER II enzyme in 15 min (Fig. 2A, lane 7). Our result showed that the cleavage efficiency was 89% under 0.05 U/μl of USER II concentration (Additional file 1: Fig. S1C, lane 2), while both 0.1 and 0.2 U/μl of USER II concentration led to 91% of cleavage rate (Additional file 1: Fig. S1C, lanes 3 and 4), which showed a similar result in Fig. 2A (lane 6). Thus, 0.1U/μl of USER II concentration (Additional file 1: Fig. S1C, lane 3) is efficient for dU cleavage compared to higher concentration (Additional file 1: Fig. S1C, lane 4).

#### Effect of dU position on adapter cleavage

dU position at the 24th of nucleotide from 5′ end is designed to increase the base paring ability by introducing cleavage. Besides, we designed two other 5′ dU adapters (dU Loop18 and dU Loop20 adapters, see Table S1 and Additional file 1: Fig. S2A) with dU at the 18th nt and 20th nt position from 5′end of the adapter to identify whether dU position affects the efficiency of USER II cleavage. Interestingly, our result showed that the cleavage rates of dU Loop18 adapter and dU Loop20 adapter were 37% and 68% (Additional file 1: Fig. S2A, lanes 2 and 4), which were much lower than the 5′ dU adapter (dU at the 24th position) designed originally (Fig. 2A), revealing that dU in the single-stranded loop might decrease the USER II cleavage rate compared to dU in the double-stranded DNA (ds DNA). To further verify this, we have also designed three 5′ adapters with dU at 27th, 32th and 34th positions (dU Stem27, dU Stem32 and dU Stem34 adapters, see Additional file 1: Table S1 and Fig. S2B) from 5′ end to identify their cleavage efficiency under 15 min of reaction time (Additional file 1: Fig. S2B). The cleavage rates of dU Stem27, dU Stem32 and dU stem34 adapters by USER II enzyme were 90%, 91% and 84%, respectively (Additional file 1: Fig. S2B, lanes 2, 4 and 6). In this way, USER II displayed higher efficiency in dsDNA than that of ssDNAs (Additional file 1: Fig. S2A, lanes 2 and 4). Therefore, we concluded that dU at 24th position of 5′ adapter works well with USER II cleavage.

### 5′ dU adapter ligation

To assess the efficiency of 5′ ligation process, a 66-nt nucleotide consists of 40 randomized nucleotides and 22-nt 3′ adapter sequence [23] in the 3′ end (N40+22, see Additional file 1: Table S1), which has been designed to mimic cDNA product after 3′ ligation and reverse transcription in the real library, and ligated to the 44-nt 5′ dU adapter to produce a 106-nt ligated product with the help of T4 DNA ligase. As we have optimized three factors including cDNA: adapter ratio, PEG 6000 concentration and PEG type with normal adapter in a previous study [23], here we only examined the effect of dU adapter concentration on the 5′ ligation efficiency with other reaction conditions remain unchanged. Four different ratios of cDNA to adapter, ranging from 1:1 to 1:10 were utilized in the 5′ ligation reaction under 17.5% PEG 6000. From the gel image (Additional file 1: Fig. S3), the ligation yield increased with the reducing DNA: adapter ratio and with 1:5 and 1:10 of DNA:adapter ratios, the ligation efficiency reached 95% (Additional file 1: Fig. S3, lanes 3 and 4). Overall, we found that 1:5 or 1:10 of DNA:adapter ratio could get the highest 5′ ligation efficiency with dU adapter.

### Purification steps before and after USER II cleavage

#### Effect of column purification on the ligated product cleavage after ligation

To investigate whether the T4 DNA ligase in the ligation reaction could affect the efficiency of dU cleavage of ligated product after USER II treatment, we performed column purification (RNA Clean & Concentrator, see the “Methods” section) after the 5′ ligation process to remove those unused substances in the ligation reaction. Even though we showed that the ligation reaction buffer and PEG 6000 concentration had no effect on the dU adapter cleavage (Additional file 1: Fig. S1B), but whether the whole ligation condition will affect the USER II cleavage rate is still unknown, so here we identified if the 5′ ligation condition affects the following USER II cleavage efficiency with or without ligation cleanup step.

In this study, the entire 106-nt ligated product, including template 44-nt dU adapter at 5′ end and 62-nt cDNA, could be cleaved into 2 fragments (85 nt and 21 nt) by USER II enzyme at dU position (Fig. 2B), therefore we evaluated the reduction rate of ligated product with and without column purification to verify if USER II cleavage is compatible with real ligation step. We found that column purification could help USER II cleave up to 86±5% of ligated product (Fig. 2B, lane 4) compared to 10±5% of cleavage rate (Fig. 2B, lane 2) without column purification after USER II treatment. The 106-nt ligated products without USER II cleavage (Fig. 2, lanes 1 and 3) were reference controls for calculation. As multiple bands showed in the gel image, making it complex to calculate the recovery rate of column purification. Thus, we used a 106-nt oligo to mimic the ligated product (Additional file 1: Table S1) and showed the recovery rate of column purification was 91±1% (Additional file 1: Fig. S4, lane 2), indicating that more than 90% product could be recovered during purification. Hence, our result showed that even in QL buffer condition, USER II cleavage is not compatible with the 5′ ligation condition due to complexity of reaction environment, and a simple column purification after 5′ ligation could help to increase the efficiency of USER II cleavage on the ligated product.

#### Effect of column purification on cleaved adapters removal after USER II cleavage

To eliminate the cleaved adapters generated from USER II cleavage after 5′ adapter ligation step, column purification (RNA Clean & Concentrator, See methods) was used to remove those small adapter fragments (23 and 21 nt), which may cause formation of primer-adapter dimer during the following PCR step, then affect the final yield of PCR product. In this study, we performed USER II cleavage on the ligated product and evaluated if the effect of column purification on the reduction rate of cleaved adapters is similar or higher than the test above. Our result showed that up to 91±1% of cleaved adapters were removed by column purification compared to the one without cleanup procedure after USER II cleavage process (Fig. 2C, lanes 3 and 4). In brief, column purification after USER II step could clear up most cleaved adapters to obtain a clean template for the following PCR amplification, and the replacement of column purification to previous gel purification step (Fig. 1A) will minimize the potential sample cross-contamination caused by gel loading and cutting, and increase the throughput of sample processing and recovery yield.

### Effect of dU adapter on PCR amplification

To determine whether the involvement of the dU adapter could increase the PCR amplification efficiency, we compared the yields of PCR product between the normal adapter and dU adapter. The primary reason to design this dU adapter is to reduce the adapter-primer dimer formation during the PCR process in the library preparation and increase the efficiency in PCR amplification. Thus, 100 ng polyA RNA input was used to generate a real cDNA template with a normal adapter or dU adapter following related rG4-seq [23] and rG4-seq 2.0 (see the “Methods” section) to perform PCR amplification experiment with increasing PCR cycles (8, 11, 14 and 17) and ran the samples on a 2% agarose gel to quantify the PCR products.

The expected PCR product (150–400 nt) was quantified and compared. Our result showed that dU adapter contributed to higher PCR efficiency than the normal adapter under the same PCR cycle (Fig. 2D). Moreover, for library preparation with 100 ng polyA-RNA input, PCR product with 11 cycles with the dU adapter (Fig. 2D, lane 7) was adequate for sequencing, while more than 17 PCR cycles were required with normal adapter (Fig. 2D, lane 4). Unspecific PCR products from 5′ dU adapter appeared at 14 and 17 cycles (Fig. 2D, lanes 8 and 9), suggesting more than 14 PCR cycles could result in over-amplification using dU adapter. The overall PCR yield was significantly increased with the dU adapter compared to the normal adapter (Fig. 2E). In conclusion, the dU adapter could help to get the required PCR product with fewer cycles than the normal adapter.

### Benchmarking of rG4-seq 2.0

**Fig. 3.**
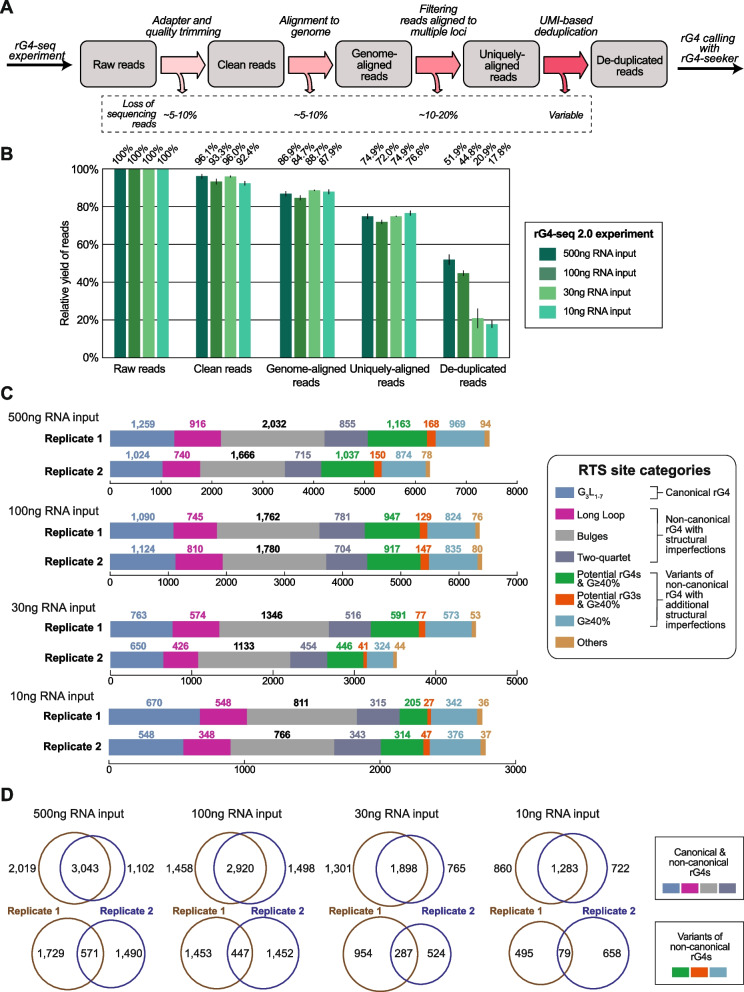
The metrics and rG4 detection outcomes of benchmarking rG4-seq 2.0 experiments. **A** Bioinformatic workflow of the pre-processing and deduplication of rG4-seq sequencing data. The estimated loss of sequencing reads in each step is highlighted. **B** Changes in the relative yield of reads in benchmarking rG4-seq 2.0 libraries through the pre-processing and deduplication steps. **C** Distribution of RTS sites detected in the benchmarking rG4-seq 2.0 experiments. RTS sites were assigned to rG4 structural classes according to their adjacent nucleotide sequences. RTS sites classified as “Others” were considered false positive detections as their adjacent nucleotide sequences do not satisfy the minimum requirements for forming quadruplex or triplex structures. **D** Reproducibility of RTS sites within biological replicates of benchmarking rG4-seq experiments. Raw data values are provided in the Additional file 8

To benchmark the performance of the rG4-seq 2.0 protocol (Additional file 1: Fig. S5), new two-replicate rG4-seq 2.0 libraries (with each replicate performed in both Li^+^ and K^+^ conditions) using variable inputs of HEK293T polyA-enriched RNA (500 ng, 100 ng, 30 ng and 10 ng per sample) were constructed and sequenced with NGS at depth of approximately 100 million read-pairs per sample. The 500-ng input library served as a baseline condition, using the RNA input amount recommended by the original rG4-seq protocol [22]. Meanwhile, the 100-ng/30-ng/10-ng input libraries served to simulate rG4-seq applications with reduced RNA inputs. To evaluate the improvements introduced by the 5′ dU adapter in a realistic scenario of rG4-seq application, a two-replicate rG4-seq 1.0 library using 100 ng of RNA input and a normal 5′ adapter was also constructed as a control group. rG4-seq 2.0 and 1.0 libraries shared the same protocol, except for the type of 5′ adapter used and the PCR cycles spent during library amplification. All rG4-seq data were then pre-processed, deduplicated using unique molecular identifiers (UMIs), and analyzed with rG4-seeker to identify rG4-induced RTS events as previously described (Fig. 3A, Additional file 1: Table S2) [24]. The workflow-enabled quantitative evaluation of the influence of RNA input amount on library complexity and rG4 identification outcome.

### rG4-seq 2.0 supports rG4 identification at variable RNA input levels

Since rG4 identification with rG4-seq is established on the capturing of RTS event from unique cDNA fragments, not all sequenced reads in a library are relevant to bioinformatic analysis – only the subset of clean reads uniquely aligned to reference genome are utilized, and the UMI-based deduplication step further eliminates reads carrying duplicate information (Fig. 3A). Reads discarded in different stages of analysis are considered as a loss in read yield as they took up sequencing throughput without contributing to rG4 identification efforts. Our previous and proposed modification to the rG4-seq protocol focused on improving read yield via reducing non-usable reads (e.g., adapter reads) and increasing the unique cDNA fragments produced (a.k.a. library complexity) per unit of RNA input.

To evaluate the yield of reads in the 4 HEK293T rG4-seq 2.0 libraries, the changes in yield through different stages of bioinformatic analysis were quantified (Fig. 3B). Results suggest the change in yield from trimming, alignment and filtering for uniquely mapped reads are consistent across the 4 libraries, where 70-80% of reads were retained through these steps (Fig. 3B, Additional file 1: Table S2). The yield figures are also on-par with normal expectations of a typical RNA-seq library [37]. On the other hand, the RNA input amount affected the yield from the deduplication step substantially: the baseline 500 ng-input library recorded a ~30% yield loss, the 100-ng input library recorded a ~40% loss, and the 30-ng/10-ng input libraries recorded ~75% loss (Fig. 3B). The observation is expected as library complexity scales proportionally with input RNA amount, such that a lower input library typically would have a higher fraction of duplicated reads at the same sequencing depth. Furthermore, as the PCR duplication rates are similar between the 500-ng input and 100-ng input libraries despite a 5-fold difference in RNA amount, indicating the 100 million reads-pair sequencing depth may be too shallow for the complexity of the 500-ng input library. In contrast, high duplicate rates in the 30-ng and 10-ng input libraries suggested that a lower sequencing depth may reduce wasted throughput and improve read yield.

To evaluate the rG4 identification outcomes of individual rG4-seq 2.0 libraries, the RTS sites were first called in a replicate-independent manner and classified by their associated rG4 motifs (Fig. 3C, Additional file 2). The reproducibility of RTS sites across replicates was then evaluated (Fig. 3D). Results showed that the 500 ng and 100 ng replicates captured a similar number of RTS sites across different rG4 motif categories (Fig. 3C), suggesting that they are equivalent in rG4 identification outcomes. Meanwhile, although the 30 ng and 10 ng replicates captured considerably fewer RTS sites, the relative ratio of RTS sites across motif categories remained similar (Fig. 3C), suggesting both libraries would have captured a smaller, random subset of RTS sites from the HEK293T transcriptome. Also, across all libraries and replicates, the false positives (RTS sites in the “Others” category) remained low at <3% (Fig. 3C). Together, the result supports that rG4-induced RTS events were accurately and specifically captured using rG4-seq 2.0, regardless of input RNA amount.

On the other hand, the overlapping of RTS sites between the biological replicates was found to be consistently ~60–70% for canonical and non-canonical rG4s (Fig. 3D) across the 4 libraries, suggesting the reduction in RNA input would not compromise the coherence of replicates. In comparison, although the overlapping rate of RTS sites from variant rG4 motifs was substantially lower (Fig. 3D), the phenomenon is not atypical as similar observations were also made when we profiled rG4s in Human HeLa cell line and Plasmodium with rG4-seq previously [24, 27]. Our studies also suggested that in comparison with canonical/non-canonical rG4s, variant rG4 motif structures had higher stochasticity, and the property was associated with their reduced reproducibility across replicate. Nevertheless, while variant motifs contained more structural imperfections than canonical and non-canonical rG4s, experimental validation suggested that these variant motifs could also fold into genuine rG4 structures [24]. Altogether, 1364 to 3623 consensus (between 2 replicates) rG4 structures were detected from 10–500 ng input rG4-seq 2.0 libraries (Additional file 1: Table S3). The union of detected rG4s totals 5,061 (Additional file 3), among which 744 rG4s were detected across all 4 libraries (Additional file 4).

### 5′ dU adapter of rG4-seq 2.0 improves library efficiency and nucleotide bias in RTS sites identification

**Fig. 4.**
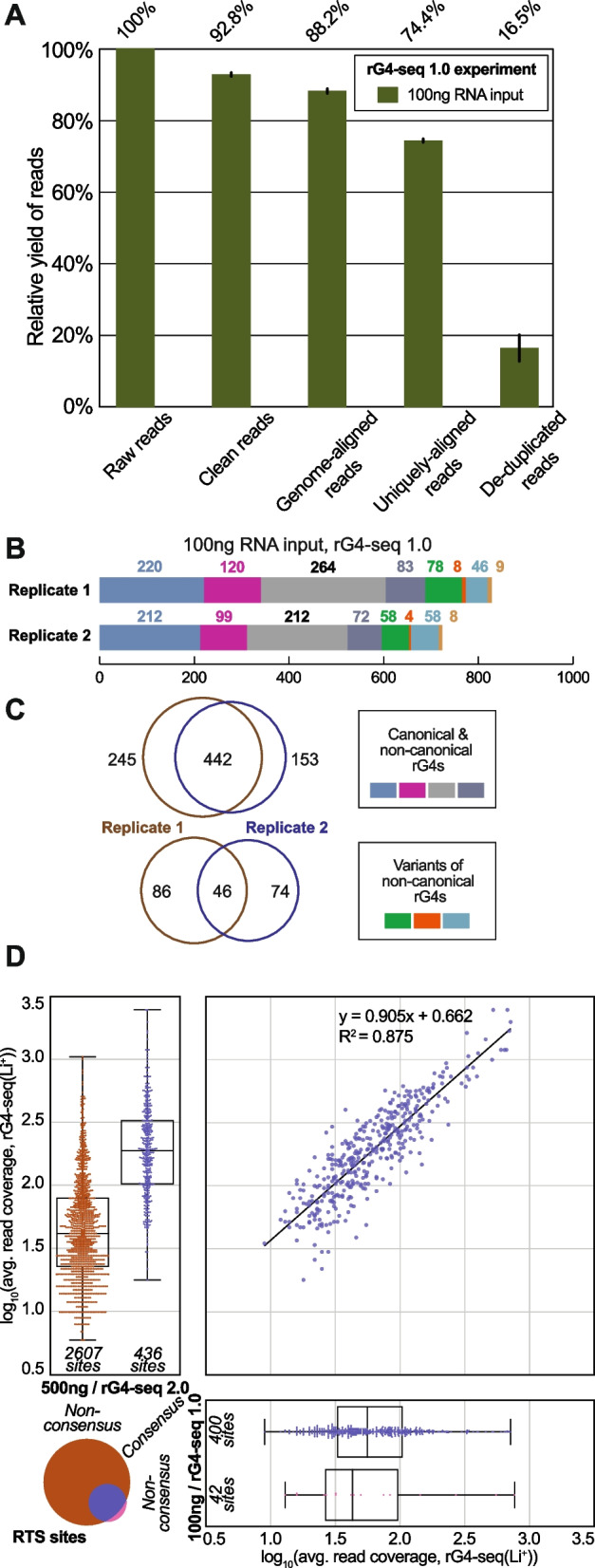
The metrics and rG4 detection outcome of the 100-ng input rG4-seq 1.0 library. **A** Changes in the relative yield of reads in the 100-ng input rG4-seq 1.0 library through the pre-processing and deduplication steps. **B** Distribution of RTS sites detected in the 100-ng input rG4-seq 1.0 experiment. RTS sites were assigned to rG4 structural classes according to their adjacent nucleotide sequences. RTS sites classified as “Others” were considered false positive detections as their adjacent nucleotide sequences do not satisfy the minimum requirements for forming quadruplex or triplex structures. **C** Reproducibility of RTS sites within biological replicates of the 100-ng input rG4-seq 1.0 experiment. **D** Intersection of consensus RTS sites detected in 500-ng input rG4-seq 2.0 experiment and 100-ng input rG4-seq 1.0 experiment. Raw data values are provided in the Additional file 8

To evaluate the effects of the new 5′ dU adapter, rG4-seq 1.0 library using 100 ng of HEK293T polyA-enriched RNA input and a normal 5′ adapter was constructed and analyzed. Results suggested that 100-ng input rG4-seq 1.0 library has a yield of ~16.5% (Fig. 4A, Additional file 1: Table S2), which is substantially lower than ~44% of the 100-ng input rG4-seq 2.0 library (Fig. 3B, Additional file 1: Table S2). Moreover, significantly fewer rG4 motifs were detected from the rG4-seq 1.0 library (Fig. 4B) in comparison to rG4-seq 2.0 libraries (Fig. 3C), suggesting the performance of rG4-seq 1.0 at 100 ng RNA input is subpar compared to rG4-seq 2.0. Nevertheless, in contrast to a reduction in rG4 detection numbers, the reproducibility of RTS sites between replicates of the rG4-seq 1.0 library (Fig. 4C) remained consistent with the observation from rG4-seq 2.0 libraries (Fig. 3D). Importantly, for the consensus set of RTS sites (canonical and non-canonical rG4s) detected, they highly overlapped (400 out of 442) with detections from the 500-ng input rG4-seq 2.0 library (Fig. 4D). This suggested despite the reduced detection numbers, rG4-seq 1.0 library indeed captured a genuine subset of rG4s in HEK293T. Altogether, 488 consensus rG4 structures (442 from canonical and non-canonical rG4s, 46 from variant rG4s) were detected in the 100-ng input rG4-seq 1.0 library (Additional file 1: Table S3). The union of detected rG4s by all rG4-seq 2.0 and 1.0 libraries totals 5085 (Additional file 5), among which 306 rG4s were detected across all 5 libraries (Additional file 6).

**Fig. 5.**
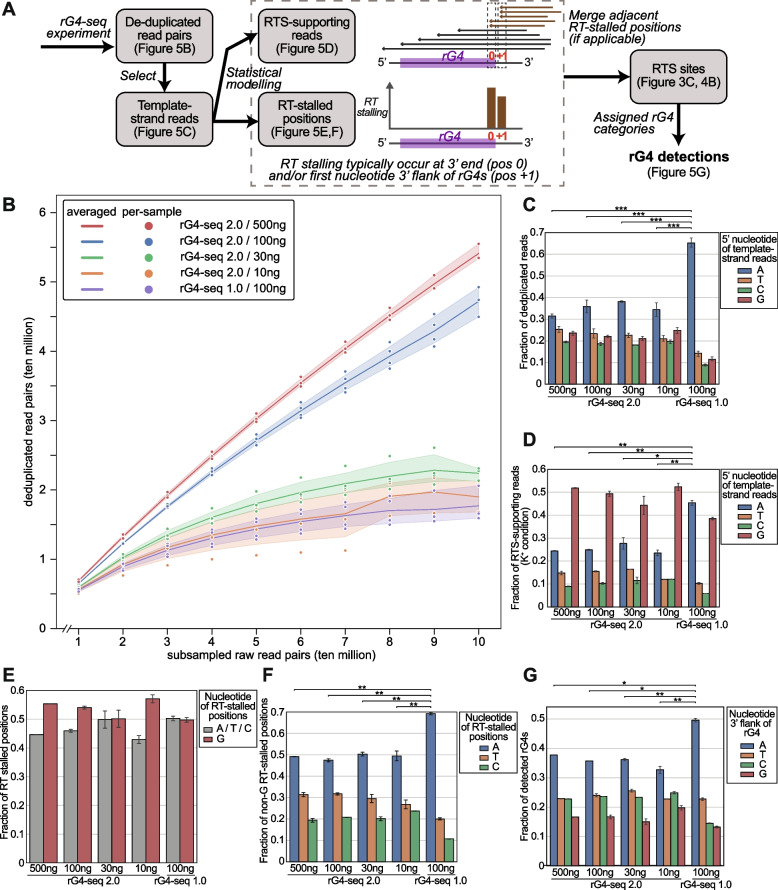
The influences of rG4-seq protocols on library efficiencies, nucleotide bias and RTS site identification outcomes. **A** Overall bioinformatic workflow of identifying RT-stalled positions, RTS sites and rG4s from rG4-seq datasets using *rG4-seeker*. Starting from deduplicated read pairs, the template-strand reads (in the same direction as RNA) were first selected. The 5′ nucleotide of template-strand reads was the position where reverse transcription stopped. Next, the K^+^ and Li^+^ libraries were compared using statistical models to identify RT-stalled positions, which were defined as the transcript positions with significantly more RT stops in K^+^ than in Li^+^. Each RT-stalled position can be supported by a varying number of template-strand reads in K^+^ libraries. The read number depended on both the abundance of the parent transcript and the strength of the RTS effect. Since each rG4 can induce an RTS effect on more than one position (typically at position 0 and/or position +1), adjacent RT-stalled positions were merged into one RTS site spanning multiple positions. Meanwhile, each singleton RT-stalled position was considered one RTS site spanning one position. RTS sites were then assigned rG4 categories based on their adjacent sequence. **B** Plot of the number of raw read pairs and deduplicated reads pairs as a function of sequencing depth for the rG4-seq 2.0 and 1.0 libraries. The variation in sequencing depths was simulated by random subsampling of the sequencing datasets. The lines and their shaded area in the plot indicated the averaged and standard deviations of the read pair numbers in respective rG4-seq libraries. Libraries with raw sequencing depth <100 million read pairs were not up-sampled and became data dropouts. **Fig. S6** in Additional file 1 showed the break-down plots for each library. **C** Distribution of the 5′ nucleotides of template-strand, deduplicated reads in rG4-seq libraries. The 5′ nucleotide was the position where reverse transcription stopped. **D** Distribution of the 5′ nucleotides of template-strand, deduplicated, RTS-supporting reads in rG4-seq libraries (K^+^ conditions only). **E**, **F** Nucleotide distribution of the RT-stalled positions detected in rG4-seq libraries, comparing **(E)** Guanine and non-Guanine positions **(F)** within non-Guanine positions. **(G)** Nucleotide distribution of the 3′ flanking nucleotide (corresponds to position +1 as described in Fig. 5A) of the detected rG4 motifs in rG4-seq libraries. While an rG4 motif always has a G nucleotide for its 3′ end (position 0), its 3′ flanking nucleotide (position +1) can be both A/T/C/G. Nucleotide bias in RT-stalled positions can favor the detection of rG4s with a specific 3′ flanking nucleotide. Asterisks represent significant differences in A-bias between two libraries in respective plots (Alexander-Govern test; ***p<0.001; **p<0.01 *p<0.05). Raw data values are provided in the Additional file 8

To explore the underlying causes of the reduced rG4 detections in the 100-ng input rG4-seq 1.0 library, we examine the aspects of rG4-seq sequencing data that are relevant to the rG4 calling process (Fig. 5A). First, the datasets were first randomly subsampled to sequencing depths between 10 and 100 million read pairs and the deduplication process was repeated. The number of deduplicated reads obtained at different sequencing depths allows for evaluating sequencing saturation, which reflects their relative library complexities. The result showed that at a depth of 100 million read pairs, 500 ng and 100-ng input rG4-seq 2.0 libraries had not yet reached saturation, while the other 3 libraries had been saturated (Fig. 5B, Additional file 1: Fig. S6). The saturation curves also indicated decreasing library complexity proportional to a reduction in RNA input (Fig. 5B, Additional file 1: Fig. S6), which has been anticipated. Importantly, the library complexity of the 100-ng input rG4-seq 1.0 library was only on par with that of the 10-ng input rG4-seq 2.0 library, showing the rG4-seq 1.0 protocol was substantially less efficient in library preparation. Given that rG4 detection requires at least moderate read coverage (typically ≥16×) around the rG4 loci to provide sufficient RTS information for statistical testing (Fig. 5A), a lower library complexity and fewer deduplicated reads give less usable RTS information and would contribute to fewer rG4s being identified.

Next, the distribution of 5′ nucleotides in the template strand of deduplicated reads was examined. Crucially, these nucleotides captured the location of RTS events (Fig. 5A). The rG4-seq 1.0 library was found to possess significantly stronger A-bias in the 5′ nucleotides (~65%) (Fig. 5C). In contrast, all 4 rG4-seq 2.0 libraries showed relatively stable distributions of 5′-most nucleotides, with ~35% As and ~20% Ts, Cs and Gs (Fig. 5C), suggesting a global A-bias in the 5′ nucleotides specific to the rG4-seq 1.0 protocol.

Furthermore, the A-bias also influenced the subset of RTS-supporting reads (Fig. 5D), as well as the nucleotide distribution of detected RT-stalled positions (Fig. 5E, F). Among the RTS-supporting reads in rG4-seq 2.0 libraries, a G-bias in the 5′ nucleotides was observed (Fig. 5D). The G-bias was anticipated as RTS effect induced by rG4s mainly occurs at the 3′ -most nucleotide of rG4 motifs (which is a G) and at the 3′ flanking nucleotide (which can A/T/C/G) (Fig. 5A). In comparison, moderate G-bias and significantly stronger, strong A-bias was observed in the rG4-seq 1.0 library (Fig. 5D), which showed that the global A-bias has been carried over. As a result, rG4-seq 1.0 library would be less efficient in capturing RTS-supporting reads (with a 5′ G nucleotide) that could indicate the presence of rG4 structures. Meanwhile, consistent across all rG4-seq 1.0 and 2.0 libraries, around half of the detected RT-stalled positions occurred on Gs, while the other half occurred on As, Ts and Cs (Fig. 5E). However, within non-G RTS events, rG4-seq 1.0 library captures significantly more RTS events on As when compared to rG4-seq 2.0 libraries (Fig. 5F). As a result, rG4-seq 1.0 library showed a significant bias in detecting rG4 that the 3′ flanking nucleotide was an A (Fig. 5G).

Together, the results indicated that compared to rG4-seq 2.0, the rG4-seq 1.0 protocol would offer lower library preparation efficiency and stronger global A-bias in the 5′ nucleotides of sequenced RNA fragments. The two issues rendered it less effective and more biased in capturing of RTS information relevant to rG4 structures at the same input RNA. The limitation surfaced when a reduced input was used, leading to fewer and more biased rG4 detections (Fig. 4B and Fig. 5F).

Given the rG4-seq 2.0 and 1.0 libraries both shared most experimental protocols, employed 5′ adapters of identical structure (except at the cleavable dU nucleotide), and incorporated UMIs for PCR deduplication, it is unlikely that their differences were caused by 5′ adapter ligation bias or PCR-duplicate reads. By elimination, the observation suggested the use of a 5′ dU adapter and adapter cleavage step in rG4-seq 2.0 improved both the library preparation efficiency and reduced nucleotide bias in capturing RTS information over the original hairpin adapter.

### rG4 identification performance is unaffected by a 5-fold RNA input reduction

To further characterize the influence of input RNA amounts on rG4 identification outcome, the consensus (from two replicates, canonical and non-canonical rG4s only) sets of RTS sites from rG4-seq 2.0 libraries were extracted and their intersections were calculated. Moreover, since rG4 detection outcomes are influenced by read coverage near rG4 motifs, the local coverage information of RTS sites was also incorporated into the analysis.

**Fig. 6.**
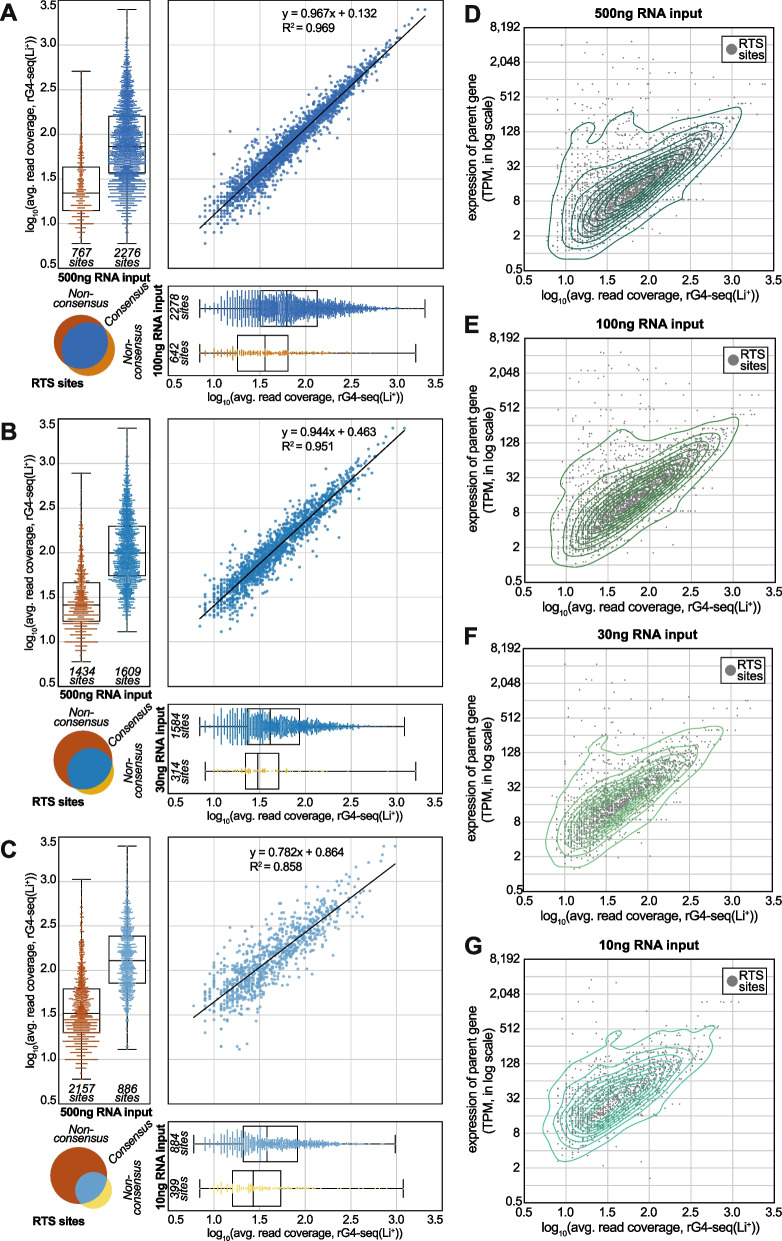
Reproducibility of RTS sites across benchmarking rG4-seq 2.0 experiments. The intersection of consensus RTS sites detected in **A** 500-ng input and 100-ng input libraries, **B** 500-ng input and 30-ng input libraries, and **C** 500-ng input and 10-ng input libraries. The Venn diagrams indicate the number of RTS sites that are agreed or non-agreed between the two libraries. The swarm plots and overlayed box plots display and contrast the differences in read coverages of agreed/non-agreed RTS sites. The scatter plots indicate the correlation and linear regression outcomes of read coverage of the RTS sites agreed between two rG4-seq libraries. **D**–**G** Average read coverages and relative abundances of the parent gene of the consensus RTS sites detected in the rG4-seq experiments. The contour lines from kernel density estimation were overlayed to help illustrate the density distribution of RTS sites

The intersection of RTS sites from the 500-ng input and 100-ng input libraries suggested that two libraries agree with each other on more than 70% of the RTS sites (Fig. 6A). Importantly, the level of agreement is proportional to read coverage: nearly all RTS sites with coverage >100× are agreed, while the non-agreed sites usually have lower coverage at between 15 and 50× (Fig. 6A). The observation is coherent with our previous findings from HeLa rG4-seq data [22], where the agreement between rG4-seq experiments is non-ideal at <32x read coverage. Nevertheless, the chance of these low-coverage RTS sites being false positives is low, since they are the consensus RTS sites supported by both biological replicates. Instead, the disagreement observed should be accounted to the stochastic nature of capturing rG4-induced RTS events with a relatively low number of sequencing reads. On the other hand, the read coverage of the agreed RTS sites was highly correlated between the two libraries (Fig. 6A): the linear regression formula suggested a slope of ~1.0 and a small intercept of 0.132, indicating the 100-ng input library contains a similar number of reads for high-coverage RTS sites, while slightly fewer reads for low-coverage RTS sites. Together, the observation supports the conclusion that the 500-ng input and 100-ng input libraries are nearly equivalent in terms of rG4 identification performances.

### Further RNA input reduction impacts the detection of low-coverage RTS sites

On the other hand, the 30-ng input and 10-ng input libraries produced different results in the intersection analysis. From the observation that respectively ~85%/~70% of RTS sites detected in the 30-ng/10-ng input library agree with the sites detected in the 500-ng input library (Fig. 6B, C), it can be confirmed that rG4 discoveries from the two libraries are genuine. However, correlation analysis indicated that consensus RTS sites in these two libraries had substantially lower read coverage than their matched counterparts in the 500-ng input library (Fig. 6B, C) – around 2x less coverage for the 30-ng input library sites, and 3–5× less for the 10-ng input library sites. Moreover, in the 30-ng input library, the linear regression formula has a slope of ~0.8, indicating the undesirable reduction in coverage tends to be amplified in the already-low coverage regions of the transcriptome. Importantly, the observation explains why the 30-ng/10-ng input library had fewer RTS detection and was unable to rediscover many of the RTS sites in the 500-ng input library: the majority of the non-rediscovered RTS sites have read coverage of 15–50× in the 500-ng input library, and the projected read coverage of these sites in a 30-ng/10-ng input scenario are either at near or at below the detection limit of rG4-seq experiments (at around 16× coverage). Together, our findings suggested that while accurate rG4 identification with rG4-seq 2.0 is possible at sub-30-ng levels of RNA input, the sensitivity for rG4s in low-coverage regions of the transcriptome might be substantially affected.

### A reference guideline for RNA input level options with rG4-seq 2.0

Finally, to outline the relationships between transcript abundances and the sensitivity for rG4s in an rG4-seq 2.0 experiment, we plotted the read coverage of the consensus RTS sites against the abundances (in TPM, transcript per million) of their parent genes for the 4 libraries (Fig. 6D–G). The plots corresponding to the 500-ng input (Fig. 6D) and 100-ng input library (Fig. 6E) showed that the majority of RTS sites were detected from parent genes of TPM 0.5–16, and the local read coverage for these RTS sites ranged between 10× and 100×. In contrast, owing to the general reduction in read coverage, RTS detections from these lower abundance genes were impacted in the 30-ng input library (Fig. 6F) while largely compromised in the 10-ng input library (Fig. 6G).

Together, our benchmarking of rG4-seq 2.0 suggested the following conclusions:

1. Regardless of input RNA amount, rG4-induced RTS events can be accurately captured to reveal the presence of rG4 structures in the HEK293T transcriptome.
2. At a 100 million read-pair sequencing depth, using 100 ng poly-A enriched RNA input per sample would be optimal using the new protocol, since an equivalent rG4 detection outcome to using a 500-ng input can be expected, and the anticipated library complexity is sufficiently high to avoid sequencing saturation.
3. Higher input RNA quantity than 100 ng would require a higher sequencing depth than 100 million read pairs to fully capture the increased library complexity.
4. Lower input RNA quantity than 100 ng would scale down the total number of rG4 detections, since it is harder to map rG4s on lower abundance genes/transcripts reliability due to increased stochasticity in RTS events and random fluctuations in sequencing read coverage. If there is no constraint on the availability of RNA material (e.g., from expandable cell lines), ample RNA input should be used to avoid risks.

In practice, if comprehensive transcriptome-wide profiling of rG4s is desired, users are advised to use 100-ng input RNA/100 million read pairs as a reference point and scale the RNA amount and sequencing throughput proportionally for optimal read yield. A very low RNA input at 10 ng levels would only be desirable if input material is limited; or if the required scope of rG4 detection is limited to higher abundance transcripts/genes, typically at >10 TPM. Meanwhile, if the detection of rG4s from rare transcripts (e.g. long non-coding RNAs) via coupling target-capture methods with rG4-seq 2.0 is desired, a practical guideline could be:

1. Relative abundances of targeted transcripts/parent genes should be ≥4 TPM post-enrichment.
2. If the abundances of targeted transcripts/parent genes post-enrichment are in the range 4–8 TPM, at least 100 ng of post-enrichment RNA should be used
3. If the abundances of targeted transcripts/parent genes post-enrichment are in the range 8–16 TPM, at least 30 ng of post-enrichment RNA should be used.
4. If the abundances of targeted transcripts/parent genes post-enrichment are in the range ≥16 TPM, at least 10 ng of post-enrichment RNA should be used.

## Conclusions

In summary, we developed the rG4-seq 2.0 protocol by incorporating the use of a dU adapter, eliminating the gel purification steps, and optimizing the experimental procedures, yielding a more user-friendly and efficient pipeline than the original rG4-seq method. We first applied the rG4-seq 2.0 to purified polyA-enriched RNA from the HEK293T cell line and generated 4 cDNA rG4-seq libraries using decreased polyA-RNA inputs (500 ng, 100 ng, 30 ng and 10 ng). All libraries showed good qualities and reproducibility with a high mapping rate of reads. rG4-induced RTS events can be accurately captured by rG4-seq 2.0 to reveal the presence of rG4 structures in the HEK293T transcriptome. Moreover, the new dU adapter in rG4-seq 2.0 improved the rG4 calling outcome by improving library preparation efficiency and mitigating a significant nucleotide bias in rG4-induced RTS site identification persistent in the old method. The improvements allowed rG4-seq to adapt to a wider range of RNA input quantities, fit in scenarios where the biological specimen has limited availability, and show better promises in capturing rG4s on low-abundance transcripts. Lastly, we outlined the relationships between transcript abundances and the sensitivity for rG4s in human transcriptome and proposed a standard recommendation, which provides the amount of RNA inputs and sequencing depth for specific rG4 studies based on the target abundance in the transcriptome.

The surveying of non-coding RNA species is one of the pending questions in transcriptome-wide RNA structure analyses. To study RNA species with low cellular abundances effectively, enrichment methods of high performance are often required, which poses a challenge for all RNA structural probing methods equally. In addition, the transcript length is also one of the considerations. While rG4-seq technology could detect rG4 in longer lncRNAs and/or pre-miRNAs, the surveying of small RNAs will be limited as the RT-stalled reads could be too short for sequencing.

rG4-seq 2.0 not only offers in vitro rG4 candidates for structural and functional characterization, but also provides resources for analyzing rG4 trans-acting factors and correlation with other RNA cis-regulating elements. The design of rG4-seq 2.0 will also offer new and important lessons for constructing cDNA libraries in wide-ranging sequencing applications, including RNA structurome profiling and other methods derived from RNA-seq.

## Methods

### Preparation of DNA oligonucleotides

All the DNA oligonucleotides used in this work were synthesized and purchased from Integrated DNA Technologies, Inc. The detailed sequences were listed in Additional file 1: Table S1. The oligos were dissolved and diluted to the desired volume with nuclease-free water accordingly. N40+22-nt DNA template was PAGE purified before use.

### USER II cleavage—the effect of reaction time

A 20 μl reaction mixture was made of 2 μl of 10 μM of either normal or dU adapter (1 μM final), 2 μl of 10× CutSmart® buffer (1× final) and 2 μl of 1U/μl USER® II enzyme (New England Biolabs, M5505S) (0.1U/μl final) and 14 μl nuclease-free water. The reaction mixture was incubated at 37°C for 0, 1, 2, 5 and 15 min. For the test of reaction time over 15 min, the same 20-μl reaction mixture was prepared and incubated at 37°C for 15, 30, 60 and 120 min. At each time point, 5 μl of the mixture was aliquoted out and added with 5 μl 2× formamide (FA) dye for quenching. The samples were subjected to gel electrophoresis afterwards (see below).

### USER II cleavage—the effect of reaction buffer

The reaction mixture was either composed with CutSmart® buffer or quick ligation reaction buffer. For the CutSmart® reaction, 1 μl of normal adapter or dU adapter (1 μM final), 1 μl of 10X CutSmart® buffer and 0 μl or 1 μl of USER® II enzyme were added to make up to 10 μl reaction volume. For the quick ligation reaction, 1 μl of normal adapter or dU adapter (1 μM final), 5 μl 2X quick ligation reaction buffer, 0, 1 or 2 µl of 50% PEG 6000 (7.5%, 12.5% or 17.5% final), 0 μl or 1 μl of USER® II enzyme and then made up to 10 μl with nuclease-free water. The reaction was incubated at 37˚C for 15 minutes. After that, 10 μl of 2X FA dye was added to each sample. Gel electrophoresis was then performed (see below).

### USER II cleavage—the effect of enzyme concentration

The reaction mixture was made of 1 μl of 10 μM dU adapter (1 μM final), 1 μl of 10X CutSmart® buffer (1× final), 0, 0.5, 1 or 2 μl of 1U/μl USER® II enzyme (New England Biolabs, M5505S) and nuclease-free water to make up to 10 μl. The reaction mixture was incubated at 37°C for 15 min. 10 μl 2× FA dye was added to each sample. Gel electrophoresis was then performed (see below).

### USER II cleavage—the effect of uracil position

The reaction mixture was composed of 1μl of 5′ dU adapter/dU Loop18 adapter/dU Loop20 adapter/dU stem27 adapter/dU stem32 adapter/dU stem34 adapter (1 μM final), 1 μl of 10× CutSmart® buffer, 7 μl nuclease-free water and 1 μl of USER® II enzyme. The reaction was incubated at 37°C for 15 min. After that, 10 μl 2× FA dye was added to the sample. Gel electrophoresis was then performed (see below).

### 5′ dU adapter ligation—the effect of DNA: adapter ratio on the ligation efficiency

The ligation reaction was carried out using Quick Ligation™ kit (New England Biolabs, M2200). A 10-μl mixture consists of 1 μl of 1 μM N40+22nt DNA (0.1 μM final), 1 μl of 5′ dU adapter with varying concentration (0.1, 0.25, 0.5 or 1 μM final), 2 μl of 50% PEG 6000, 1 μl of Quick ligase in 1× Quick ligation buffer. The reaction condition was 2 h at 37°C and 10 min at 95°C for inactivation followed by adding 10 μl 2× FA dye. The samples were subjected to gel electrophoresis (See below).

### Purification steps—the effect of column purification on the USER II cleavage after ligation

5′ adapter ligation was performed with 5 pmol DNA and 50 pmol dU adapter input in the 20-μl volume reaction. After 2-h incubation at 37 °C and 10 min at 95°C, for reactions without column purification, 5 μl of reaction was taken out followed by adding 5 μl nuclease-free and 10 μl 2× FA dye for reference. Another 5 μl of reaction was directly added with 4 μl nuclease-free water and 1 μl of USER® II enzyme and incubated at 37°C for 15 min. For reaction with column purification, 10 μl of the ligated product was added to 40 μl nuclease-free water before performing the column purification with RNA Clean & Concentrator™ (Zymo Research, R1016). After that, 10 μl nuclease-free water was eluted. 5 μl was taken out and added with 5 μl nuclease-free water followed by 10 μl 2X FA dye as reference. The other 5 μl reaction was mixed with 3 μl nuclease-free water, 1 μl CutSmart® buffer and 1 μl of USER® II enzyme, and then incubated at 37°C for 15 min and quenched with 2× FA dye. The samples were subjected to gel electrophoresis (See below).

To test the recovery rate of column purification on the ligated product, 20-μl 106-nt mimic ligated product (0.25 μM final) was prepared. Ten microliters was taken out for column purification with RNA Clean & Concentrator™ (Zymo Research, R1016) and eluted with 10 μl of nuclease-free water. The other 10 μl reaction was used for reference. each reaction was quenched with 2X FA dye. The samples were subjected to gel electrophoresis (see below).

### Purification steps—the effect of column purification on the cleaved adapters removal after USER II cleavage

To perform excess adapter removal after USER II cleavage, 20 μl of 5′ adapter ligation and column purification after ligation were stated above. Eluted the sample with 16 μl nuclease-free water followed by 2 μl CutSmart® buffer and 2 μl USER® II treatment. After 15 min incubation at 37°C, take out 10 μl and add 40 μl nuclease-free for second column purification (Zymo Research, R1016) followed by elution with 10 μl nuclease-free water. Another 10 μl sample was saved for reference control. For size references, nuclease-free water was added to replace the dU adapter or N40+22 DNA template in preparation of 5′ adapter ligation reaction. Each reaction was quenched with 10 μl 2× FA dye and gel electrophoresis was performed. (see below).

### Denaturing polyacrylamide gel electrophoresis (PAGE)

Samples were heated at 95°C for 3 min before loading into the gel. Each sample (2 μl) was then loaded into the pre-heated (300 V for 20 min) 8.3 M urea-containing 10% polyacrylamide-urea denaturing gel. The gel electrophoresis was performed at 300 V for 25 min in 1X TBE buffer.

To visualize the nucleic acids, SYBR Gold (Thermo Fisher Scientific, S11494) with 1:10,000 dilution was used to stain the gel after electrophoresis. The gel was then soaked into the 1X SYBR Gold staining solution for 5 min and then directly scanned by Bio-rad ChemiDoc^TM^ Touch Imaging System under SYBR Gold mode (UV detection) and the gel image was analysed.

### PCR amplification

One hundred-nanogram polyA-enriched RNAs from HEK 293T cell were used to generate cDNA for PCR amplification using a normal adapter following rG4-seq [23] and 5′ dU adapter (see library preparation method below). The PCR reaction included 4 μl cDNA template (from either normal or 5′ dU adapter sample), 0.5 μl of 10 μM PCR forward primer (0.5 μM final), 0.5 μl of 10 μM PCR reverse primer (0.5 μM final), and 5 μl of 2X KAPA HiFi HotStart Ready Mix (KAPA Biosystems, KK2602).

The PCR program was stated below: 95 °C, 3 min; (98 °C, 20 s; 68 °C, 15 s; 72 °C, 40 s), 72 °C, 1.5 min. The PCR amplifications were conducted in 4 different cycles, i.e. 8, 11, 14, and 17. After the amplification, the samples were subjected to 2% agarose gel electrophoresis at 120V for 60 min. The gel image was obtained for further analysis.

### Data processing

Gel bands were background-corrected and quantified by the Image Lab™ 6.0.1 software.

A. Uncleaved adapter
B. Cleaved adapter
C. N40+22-nt DNA template band
D. Ligated product without USER II treatment
E. Ligated product after USER II treatment
F. 106-nt mimic ligated adapter band without column purification as reference
G. 106-nt mimic ligated adapter band with column purification
H. Cleaved adapters before column purification as reference
I. Cleaved adapter after column purification

Cleavage rate

1 $$\textrm{Cleavage}\ \textrm{rate}=\frac{\textrm{B}}{\textrm{A}+\textrm{B}}\times 100\%$$

5′ adapter ligation efficiency

2 $$\textrm{Ligation}\ \textrm{efficiency}=\frac{\textrm{D}}{\textrm{C}+\textrm{D}}\times 100\%$$

Reduction rate of ligated product

3 $$\textrm{Reduction}\ \textrm{rate}\ \textrm{of}\ \textrm{ligated}\ \textrm{product}=\left(1-\frac{\textrm{E}}{\textrm{D}}\right)\times 100\%$$

Recovery rate of column purification

4 $$\textrm{Recovery}\ \textrm{rate}\ \textrm{of}\ \textrm{column}\ \textrm{purification}=\frac{\textrm{G}}{\textrm{F}}\times 100\%$$

Cleaved adapters removal rate

5 $$\textrm{Cleaved}\ \textrm{adapters}\ \textrm{removal}\ \textrm{rate}=\left(1-\frac{\textrm{I}}{\textrm{H}}\right)\times 100\%$$

### High-throughput library preparation

Authenticated HEK293T cells (ATCC) without mycoplasma contamination were grown in DMEM (Thermo Fisher Scientific, 10569044) media with 10% heat-inactivated FBS (Thermo Fisher Scientific, 10270106) and 1X antibiotic antimycotic (Thermo Fisher Scientific, 15240062) at 37 °C with 5% CO_2_. Total RNA was extracted from the pelleted cells using RNeasy Plus Mini Kit (Qiagen, 74136).

PolyA RNAs were purified using the Poly(A) PuristMAG Kit (Thermo Fisher Scientific, AM1922). After 2 rounds of selection, the corresponding amount of polyA RNA was incubated with 1× fragmentation buffer at 95 °C for 1 min to generate ~300 nt of RNA fragment followed by column purification (Zymo Research, R1016) as described before [23]. Next, 3′ dephosphorylation was conducted at 37 °C for 30 min including 7 μl sample after fragmentation, 1 μl rSAP enzyme (New England Biolabs, M0371L) and 1 μl PNK enzyme (New England Biolabs, M0201S) in 1× T4 PNK buffer. Then 3′-adapter ligation was performed at 25°C for 1 h by adding 1-μl 3′ adapter (with 1:10 molar ratio of RNA to 3′ adapter), 7-μl PEG 8000 (17.5% final) and 1 μl of T4 RNA ligase 2 KQ (New England Biolabs, M0373L) in 1X T4 RNA ligase buffer. After 3′ adapter ligation, added 1 μl RecJf (New England Biolabs, M0331) and 1 μl 5′ deadenylase (New England Biolabs, M0331) into the 20 μl ligation reaction for incubation with 30 min at 30°C and 3 min at 65°C to inactivate the enzymes followed by column purification (Zymo Research, R1016). Before the reverse transcription (RT), each sample was then equally separated into 2 reactions. Each reaction includes 12 μl of sample, 1 μl reverse primer (with 1:2.5 molar ratio of RNA to reverse primer), and 6 μl Li^+^ or K^+^ RT buffer [23]. RT process was described in a previous study [23]. After RT, cDNA was purified for further 5′ adapter ligation. At this step, 1 μl 5′ dU adapter (with 1:10 molar ratio of cDNA to 5′ adapter) was mixed with 7 μl eluted cDNA sample and then was heated at 95 °C for 3 min, 60 °C for 1 min and 25 °C for 2 min. After the incubation, 10-μl 2× Quick T4 ligase buffer and 2-μl Quick T4 DNA ligase (New England Biolabs, M2200L) were added to the reaction and incubate at 37 °C for overnight. The ligated products (≥200 nt) were then purified by RNA Clean & Concentrator (Zymo Research, R1016). Subsequent steps including dU adapter cleavage with CutSmart® buffer and column purification were stated in detail above. After the PCR cycle test (usually PCR cycle with 5-8 ng/μl of target product determined by Agilent Technologies 4200 TapeStation is appropriate), real PCR reaction of each sample including 8 μl purified ligated product after dU cleavage, 1 μl 10 μM forward primer, 1 μl 10 μM reverse primer with different 6-nt index sequence (barcode) and 10 μl 2X KAPA HiFi HotStart ReadyMix (KAPA Biosystems, KK2602), was performed using 95 °C, 3 min, appropriate cycles of each temperature step (98 °C, 20 s; 65 °C, 15 s; 72 °C, 40 s) based on the PCR test result. Then PCR products with loading dye were purified with 2% agarose gel and sizes 150–400 bp were sliced and extracted (Zymo research, D4008). The following steps were performed similar as previously described [23]. The rG4-seq 1.0 libraries with 100 ng polyA-enriched RNA input were performed based on the previous method [23]. Library quantification was obtained by qPCR (KAPA, KK4824). Each replicate of 500 ng and 100 ng of quantified samples was then pooled and subjected to next-generation sequencing (GENEWIZ, Azenta Life Sciences) on the Illumina Hiseq System, in a 2×150-bp paired-end configuration for 1 lane (with 1% Phix). The same procedure was performed for 30-ng and 10-ng input samples.

### Sequencing data processing and bioinformatic analysis

UMI barcodes in raw sequencing reads were first extracted using UMI-tools [38]. Sequencing reads were then adapter-trimmed and quality-trimmed using cutadapt [39]. Trimmed reads were aligned to GRCh38/hg38 human reference genome using a STAR aligner [40]. Uniquely aligned reads were deduplicated based on UMI information using UMI-tools [38]. Deduplicated and uniquely aligned reads were subjected to an rG4-seeker pipeline [24] to identify RTS sites and rG4 motifs at default settings. Ensembl 97 gene annotation [41] was used to define the transcriptomic regions. Transcript abundances (in TPM) were quantified using Kallisto [42] employing all aligned reads and were then summed to obtain gene abundances. Downstream analysis to intersect RTS sites detected in respective rG4-seq libraries and to match RTS sites with parent gene abundances were conducted with Python, where two RTS sites with <5nt distance on genomic coordinates were considered overlapping. Consensus lists of rG4s detected across rG4-seq 2.0 and rG4-seq 2.0/1.0 libraries were obtained with iterative runs of bedtools intersect [43] and using 500-ng input rG4-seq 2.0 library as the basis. Union lists of rG4s detected across rG4-seq 2.0 and rG4-seq 1.0 libraries were obtained using bedtools merge [43], where adjacent/overlapping rG4 motifs on the same strand from different libraries were merged.

To evaluate sequencing saturation, raw reads were random to sequencing depths between 10-100 million read pairs at the multiples of 10 million read pairs. Subsampling was omitted if the target depth is higher than the number of reads available. Uniquely aligned reads were randomly subsampled by selecting reads whose read name matched the set of subsampled raw reads. PCR deduplication was conducted using the subsampled uniquely aligned reads.

The commands and configurations of the respective bioinformatic tools used were provided in Additional file 7.

## Supplementary Information

**Additional file 1: Fig. S1.** Effect of different conditions on the 5’ dU adapter cleavage. (A) The effect of reaction time over 15min on 5’ dU adapter cleavage. The figure shows the cleavage of dU adapter (lanes 2-5) with USER II at 0, 15, 30, 60 and 120 min. There is no cleavage observed at 0 min (lane 1). The cleavage rate reaches over 99% in all reaction time from 15 minutes to 2 hours (lanes 2-5). (B) The effect of reaction buffer and different percentage of PEG 6000 on the 5’ dU adapter cleavage. The figure shows the dU cleavage under CutSmart (CS) buffer or Quick Ligase (QL) Buffer (Lanes 3-6) and with the percentage of PEG 6000 ranging from 7.5%-17.5% (Lanes 4-6). No cleavage is observed without USER II addition regardless reaction buffer type (Lanes 1-2). The cleavage rates are 97±2% under both CS buffer and QL buffer (Lane 3 and 4). From 7.5%-17.5% PEG6000 concentration under QL buffer, the % of adapter cleavage are 97-98% (Lanes 4-6), which are similar with the one under CS buffer (Lane 3). (C) Effect of enzyme concentration on 5’ dU adapter cleavage. Different enzyme concentrations from 0-0.2 U/μl are tested to evaluate the dU adapter cleavage rate. It reaches highest efficiency with 0.1 U/μl (Lane 3). Higher USER II concentration could not increase the cleavage rate (Lane 4). The DNA size marker indicates the fragment size. Equation 1 (See Methods) is used for calculation. The errors showed are standard deviation. nt=nucleotide. Three biological replicates are performed in this figure. Raw data values are provided in the Additional file 8. **Fig. S2.** Effect of uracil position on adapter cleavage. (A) dU cleavage on dU loop adapters. No cleavage is observed on the dU Loop18 adapter and dU Loop20 adapter without USER II treatment (Lanes 1 and 3). the cleavage rate of dU Loop18 adapter is 37±1% (Lane 2), and for dU Loop20 adapter, the cleavage rate is 68±1% (Lane 4), which showed lower cleavage efficacy than 5’ dU adapter (Figure 2, lane 7). (B) dU cleavage on dU stem adapters. The 3 stem adapters without USER II treatment were used as negative controls (Lanes 1, 3 and 5). The dU cleavage rates are 90±1%, 91±1% and 84±1% for Stem27 adapter, dU Stem32 adapter and dU Stem34 adapter (Lanes 2, 4 and 6), individually, which are much higher than that of dU Loop adapters (Lanes 2 and 4) showed in (A). The DNA size marker indicates the fragment size. Equation 1 (See Methods) is used for calculation. The errors showed are standard deviation. nt=nucleotide. Three biological replicates are performed in this figure. Raw data values are provided in the Additional file 8. **Fig. S3.** The effect of dU adapter concentration on ligation efficiency. The 106-nt ligated product is generated with varying cDNA: adapter ratio, from 1:1 to 1:10. The ligation yield enhanced with the decreasing DNA: adapter ratio and it reaches to the highest in 1:5 and 1:10 of DNA:adapter ratios (Lanes 3 and 4). Equation 2 is used for the yield% calculation (see Methods). The errors showed are standard deviation. nt=nucleotide. Three biological replicates are performed in this figure. Raw data values are provided in the Additional file 8. **Fig. S4.** Effect of column purification on the 106-nt mimic ligated product. The column recovery rate on the ligated product is 91±1% (Lane 2). The band of 106-nt mimic ligated product without column purification (Lane 1) is defined as reference to calculate the recovery rate. Equation 4 is used for the recovery rate of calculation (see Methods). The error showed are standard deviation. nt=nucleotide. Three biological replicates are performed in this figure. Raw data values are provided in the Additional file 8. **Fig. S5.** Experimental flowchart of RNA G-quadruplex structure sequencing 2.0 (rG4-seq 2.0). PolyA-enriched RNA (polyA-RNA) was obtained after 2 rounds of enrichment from total RNA. Around 250 nt random RNA fragments were generated during fragmentation step. Dephosphorylation reaction replaces the 3′-P of RNA with 3′-OH for the following 3’ adapter ligation. 3′ adapter is then ligated to RNA fragment followed by excess adapter digestion and removal. Reverse transcription (RT) is performed under either Li+ or K+ condition. After the RT, 5′dU adapter is then ligated to cDNA followed by ligation cleanup. USER II enzyme cleaves the dU in the ligated product followed by the excess adapters cleanup. PCR amplification is performed afterwards to produce DNA library. The purified cDNA library is then sent for next-generation sequencing (NGS) followed by bioinformatic analysis. **Fig. S6.** The variation in sequencing depths was simulated by random subsampling the sequencing datasets. Libraries with raw sequencing depth <100 million read pairs were not up-sampled and became data dropouts. Raw data values are provided in the Additional file 8. **Table S1.** DNA oligonucleotides used in this study. **Table S2.** Number of usable sequencing reads in respective bioinformatic analysis steps. **Table S3.** Number of rG4 motifs identified in rG4-seq libraries.

**Additional file 2.** Details of identified rG4 in each rG4-seq library.

**Additional file 3.** The list of union rG4s across rG4-seq 2.0 libraries.

**Additional file 4.** The list of consensus rG4s across rG4-seq 2.0 libraries.

**Additional file 5.** The list of union rG4s across rG4-seq 2.0 and 1.0 libraries.

**Additional file 6.** The list of consensus rG4s across rG4-seq 2.0 and 1.0 libraries.

**Additional file 7.** The commands and configurations of the respective tools used in the bioinformatic analysis.

**Additional file 8.** Raw data of Figures.

## Data Availability

All data generated or analysed during this study are included in this published article, its supplementary information files and publicly available repositories. Sequencing datasets generated in this study are available in the NCBI SRA repository under the accession number PRJNA728962.
