# Supplementary material for: Enhanced transcriptome-wide RNA G-quadruplex sequencing for low RNA input samples with rG4-seq 2.0
Source: BMC Biol. 2022 Nov 13;20:257. doi: 10.1186/s12915-022-01448-3 (PMC9661767; doi:10.1186/s12915-022-01448-3)
Supplement: Supplementary file 1 — Additional file 1: Fig. S1. Effect of different conditions on the 5’ dU adapter cleavage. (A) The effect of reaction time over 15min on 5’ dU adapter cleavage. The figure shows the cleavage of dU adapter (lanes 2-5) with USER II at 0, 15, 30, 60 and 120 min. There is no cleavage observed at 0 min (lane 1). The cleavage rate reaches over 99% in all reaction time from 15 minutes to 2 hours (lanes 2-5). (B) The effect of reaction buffer and different percentage of PEG 6000 on the 5’ dU adapter cleavage. The figure shows the dU cleavage under CutSmart (CS) buffer or Quick Ligase (QL) Buffer (Lanes 3-6) and with the percentage of PEG 6000 ranging from 7.5%-17.5% (Lanes 4-6). No cleavage is observed without USER II addition regardless reaction buffer type (Lanes 1-2). The cleavage rates are 97±2% under both CS buffer and QL buffer (Lane 3 and 4). From 7.5%-17.5% PEG6000 concentration under QL buffer, the % of adapter cleavage are 97-98% (Lanes 4-6), which are similar with the one under CS buffer (Lane 3). (C) Effect of enzyme concentration on 5’ dU adapter cleavage. Different enzyme concentrations from 0-0.2 U/μl are tested to evaluate the dU adapter cleavage rate. It reaches highest efficiency with 0.1 U/μl (Lane 3). Higher USER II concentration could not increase the cleavage rate (Lane 4). The DNA size marker indicates the fragment size. Equation 1 (See Methods) is used for calculation. The errors showed are standard deviation. nt=nucleotide. Three biological replicates are performed in this figure. Raw data values are provided in the Additional file 8. Fig. S2. Effect of uracil position on adapter cleavage. (A) dU cleavage on dU loop adapters. No cleavage is observed on the dU Loop18 adapter and dU Loop20 adapter without USER II treatment (Lanes 1 and 3). the cleavage rate of dU Loop18 adapter is 37±1% (Lane 2), and for dU Loop20 adapter, the cleavage rate is 68±1% (Lane 4), which showed lower cleavage efficacy than 5’ dU adapter (Figure 2, lane 7). (B) dU cleavage on [file 12915_2022_1448_MOESM1_ESM.pdf]

Supporting Information

Enhanced transcriptome-wide RNA G-quadruplex sequencing for low RNA input samples with rG4-seq 2.0

Jieyu Zhao[1,†], Eugene Yui-Ching Chow[2,†], Pui Yan Yeung[1], Qiangfeng Cliff Zhang[3], Ting-Fung Chan[2,*], and Chun Kit Kwok[1,4,*]

[1] Department of Chemistry, and State Key Laboratory of Marine Pollution, City University of Hong Kong, Kowloon Tong, Hong Kong SAR, China

[2] School of Life Sciences, and State Key Laboratory of Agrobiotechnology, Chinese University of Hong Kong, Shatin, Hong Kong SAR, China

[3] MOE Key Laboratory of Bioinformatics, Center for Synthetic and Systems Biology, Tsinghua- Peking Joint Center for Life Sciences, School of Life Sciences, Tsinghua University, Beijing 100084, China

[4] Shenzhen Research Institute of City University of Hong Kong, Shenzhen, China

* To whom correspondence should be addressed. Chun Kit Kwok. Tel: +852 3442 6858; Fax: +852 3442 0522; Email: ckkwok42@cityu.edu.hk or Ting-Fung Chan. Tel: +852 3943 6876; Fax: +852 2603 7246; Email: tf.chan@cuhk.edu.hk

†The authors wish it to be known that, in their opinion, the first two authors should be regarded as Joint First Authors.

Table of Contents

Figure S1. Effect of different conditions on the 5' dU adapter cleavage

Figure S2. Effect of uracil position on adapter cleavage

Figure S3. The effect of dU adapter concentration on ligation efficiency

Figure S4. Effect of column purification on the 106-nt mimic ligated product

Figure S5. Experimental flowchart of RNA G-quadruplex structure sequencing 2.0 (rG4-seq 2.0)

Figure S6. Breakdown plots of the number of raw read pairs and deduplicated reads pairs as a function of sequencing depth for the rG4-seq 2.0 and 1.0 libraries

Table S1. DNA oligonucleotides used in this study

Table S2. Number of usable sequencing reads in respective bioinformatic analysis steps

Table S3. Number of rG4 motifs identified in rG4-seq libraries

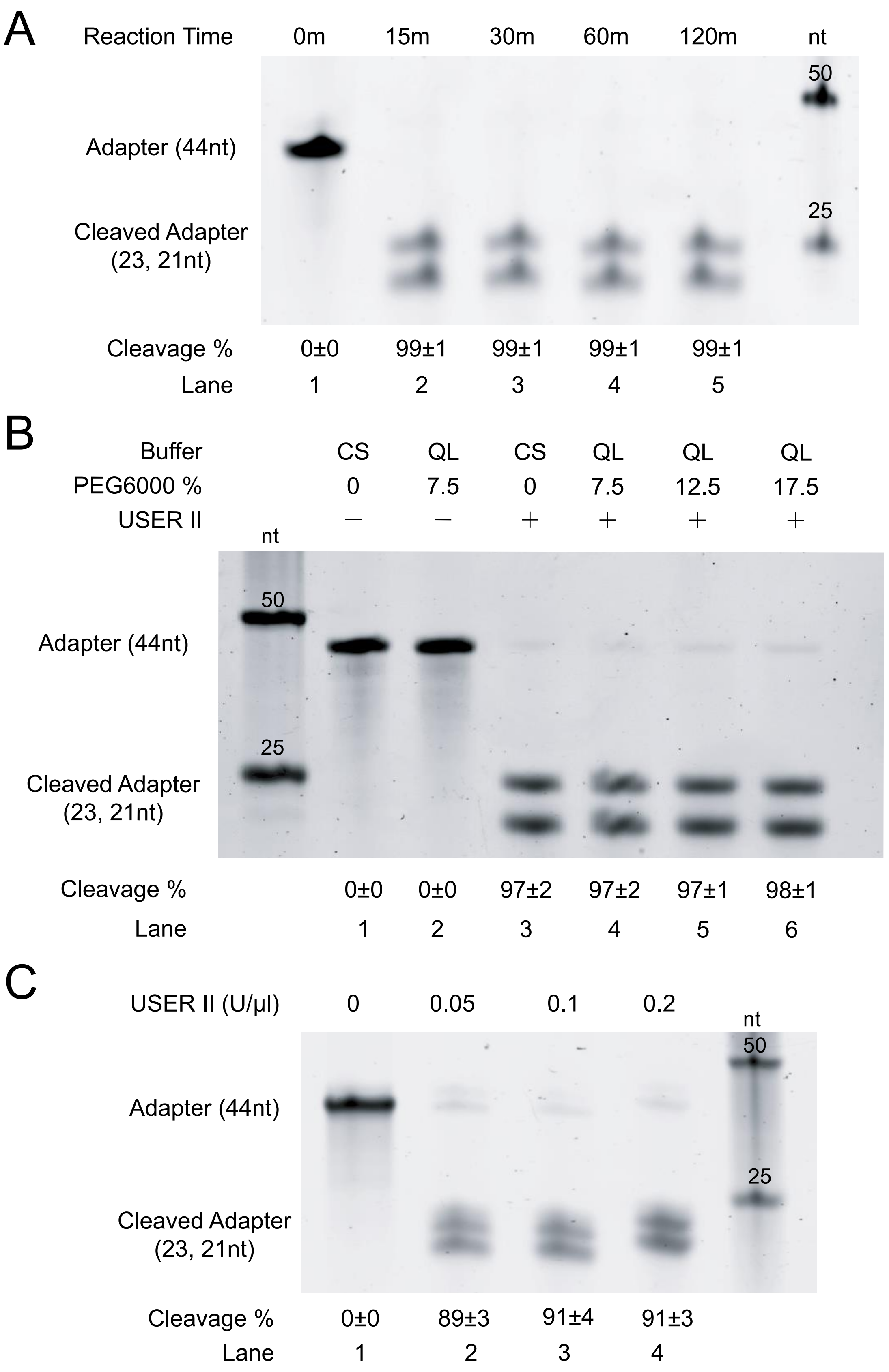

Figure S1. Effect of different conditions on the 5’ dU adapter cleavage.

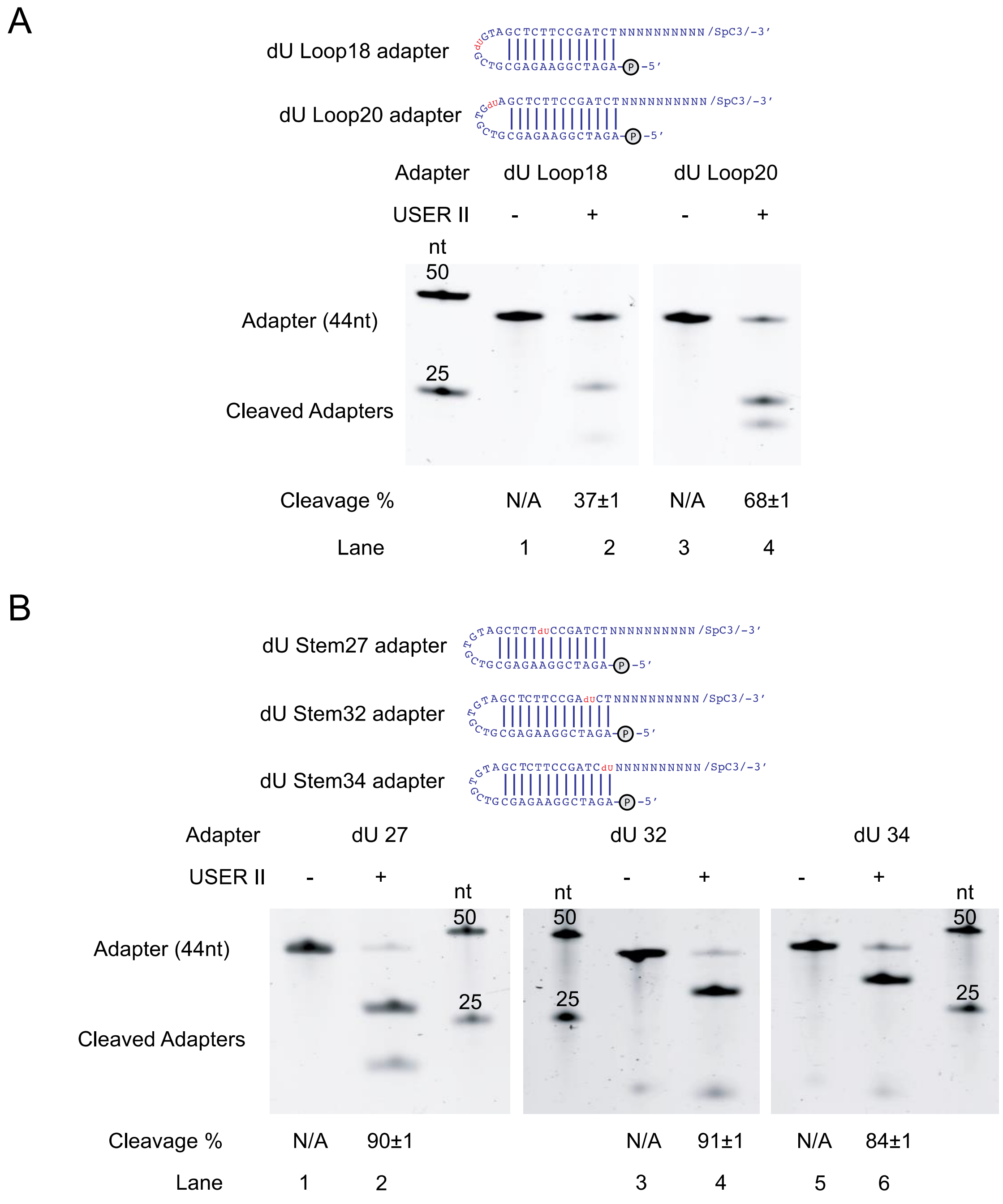

Figure S2. Effect of uracil position on adapter cleavage.

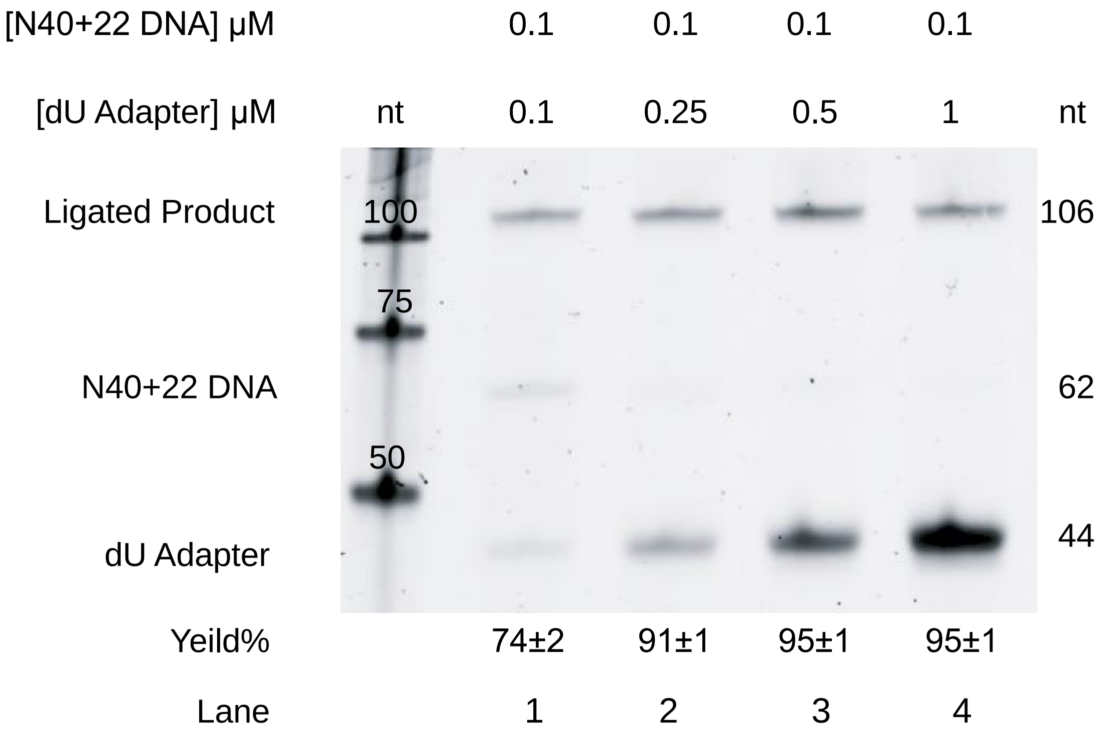

Figure S3. The effect of dU adapter concentration on ligation efficiency.

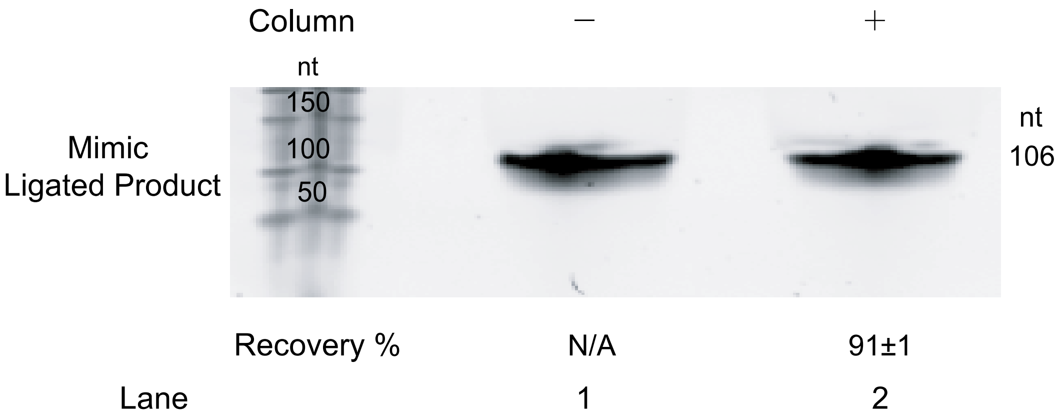

Figure S4. Effect of column purification on the 106-nt mimic ligated product.

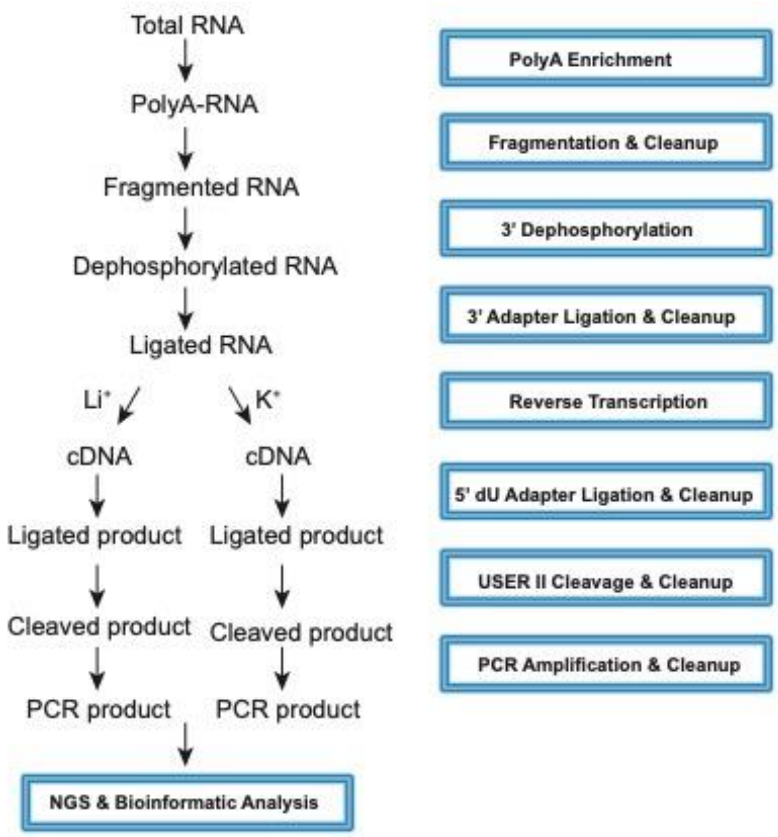

Figure S5. Experimental flowchart of RNA G-quadruplex structure sequencing 2.0 (rG4-seq 2.0).

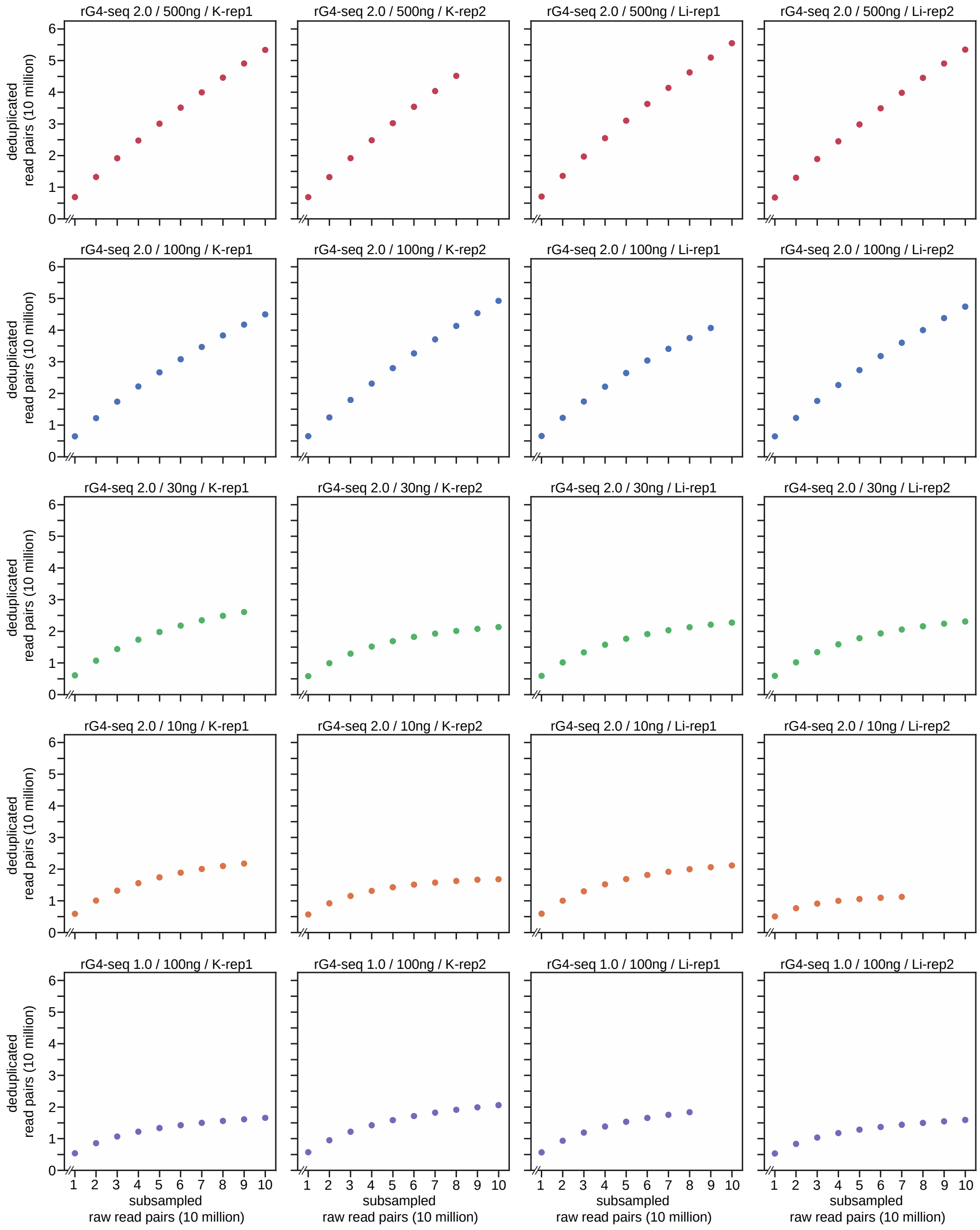

Figure S6. Breakdown plots of the number of raw read pairs and deduplicated reads pairs as a function of sequencing depth for the rG4-seq 2.0 and 1.0 libraries.

Table S1. DNA oligonucleotides used in this study.

Name of Oligo	Reaction	Length (nt)	Sequence	Modification
Normal 5' adapter	dU Cleavage	44	5'-/5Phos/AGATCGGAAGAGCGTCGTGTAGCTCTTCCGATCTN$_{10}$/3SpC3/-3'	5'-P; 3'-SpC3
5' dU Adapter	dU Cleavage	44	5'-/5Phos/AGATCGGAAGAGCGTCGTGTAGC/dU/CTTCCGATCTN$_{10}$/3SpC3/-3'	5'-P; 3'-SpC3
dU Loop18 adapter	dU Cleavage	44	5'-/5Phos/AGATCGGAAGAGCGTCG/dU/GTAGCTCTTCCGATCTN$_{10}$/3SpC3/-3'	5'-P; 3'-SpC3
dU Loop20 adapter	dU Cleavage	44	5'-/5Phos/AGATCGGAAGAGCGTCGTG/dU/AGCTCTTCCGATCTN$_{10}$/3SpC3/-3'	5'-P; 3'-SpC3
dU Stem27 adapter	dU Cleavage	44	5'-/5Phos/AGATCGGAAGAGCGTCGTGTAGCTCT/dU/CCGATCTN$_{10}$/3SpC3/-3'	5'-P; 3'-SpC3
dU Stem32 adapter	dU Cleavage	44	5'-/5Phos/AGATCGGAAGAGCGTCGTGTAGCTCTTCCGA/dU/CTN$_{10}$/3SpC3/-3'	5'-P; 3'-SpC3
dU Stem34 adapter	dU Cleavage	44	5'-/5Phos/AGATCGGAAGAGCGTCGTGTAGCTCTTCCGATC/dU/N$_{10}$/3SpC3/-3'	5'-P; 3'-SpC3
N40+22nt DNA	5' Adapter Ligation	62	5'-N$_{40}$TCTAGCCTTCTCGTGTGCAGAC-3'	5'-OH; 3'-OH

106-nt mimic ligated product	Column purification recovery	106	5’-CAGACGTGTGCTCTTCCGATCTNNNNNNNNNNNNNNNNNNNNNNNNNNNNNNNNNNNNNNNNAGATCGGAAGAGCGTCGTGTAGCTCTTCCGATCTNNNNNNNNNN-3’	5'-OH; 3'-OH
Forward Primer	PCR Amplification	58	5’-AATGATACGGCGACCACCGAGATCTACACTCTTTCCCTACACGACGCTCTTCCGATCT-3’	5'-OH; 3'-OH
Reverse Primer	PCR Amplification	58+6	5’-CAAGCAGAAGACGGCATACGAGAT-(6 nt index_seq)-GTGACTGGAGTTCAGACGTGTGCTCTTCCGATCT-3’	5'-OH; 3'-OH

Table S2. Number of usable sequencing reads in respective bioinformatic analysis steps.

**rG4-seq 2.0 Library**	**Raw read pairs**	**Clean read pairs**	**Mapped read pairs**	**Uniquely-aligned read pairs**	**Deduplicated read pairs**
500ng-K-rep1	137,398,397	133,167,732	120,159,372	103,560,827	68,125,003
500ng-K-rep2	88,985,820	84,838,879	77,011,859	66,541,953	49,331,989
500ng-Li-rep1	166,406,649	162,151,063	147,114,022	127,195,204	82,133,712
500ng-Li-rep2	100,566,437	95,288,401	85,623,158	73,466,003	53,703,838
100ng-K-rep1	110,410,923	103,333,453	93,679,546	79,544,932	48,137,468
100ng-K-rep2	122,039,081	112,433,798	102,468,376	87,309,462	57,260,162
100ng-Li-rep1	97,102,071	92,672,652	83,919,534	71,310,823	42,817,726
100ng-Li-rep2	126,122,160	116,111,852	105,244,652	89,419,592	56,094,552
30ng-K-rep1	93,804,399	89,440,824	83,022,991	70,261,384	26,488,259
30ng-K-rep2	165,418,442	158,976,261	146,362,570	123,440,346	23,378,203
30ng-Li-rep1	121,866,093	117,261,135	108,314,277	91,499,372	23,861,539
30ng-Li-rep2	109,402,650	105,156,448	97,080,342	81,955,634	23,645,790
10ng-K-rep1	114,115,611	104,060,606	98,905,474	86,175,293	23,158,515
10ng-K-rep2	94,216,637	88,586,738	84,416,825	74,012,659	16,809,716
10ng-Li-rep1	124,962,015	115,322,248	110,028,002	95,541,621	22,152,141
10ng-Li-rep2	74,104,271	68,206,790	64,690,588	56,222,720	11,346,437
**rG4-seq 1.0 Library**	**Raw read pairs**	**Clean read pairs**	**Mapped read pairs**	**Uniquely-aligned read pairs**	**Deduplicated read pairs**
100ng-K-rep1	145,927,820	134,863,585	128757741	107,794,352	18,102,642
100ng-K-rep2	130,054,116	120,094,560	113843448	96,142,554	22,195,285
100ng-Li-rep1	83,877,386	78,619,804	74856135	62,552,149	18,651,014
100ng-Li-rep2	115,133,457	106,623,017	100956422	86,458,904	16,487,931

Table S3. Number of rG4 motifs identified in rG4-seq libraries

	HEK293-rG4-seq-2.0-10ng			HEK293-rG4-seq-2.0-30ng			HEK293-rG4-seq-2.0-100ng			HEK293-rG4-seq-2.0-500ng			HEK293-rG4-seq-1.0-100ng		
rG4 motifs	**K-rep1**	**K-rep2**	**Con-sensus**	**K-rep1**	**K-rep2**	**Con-sensus**	**K-rep1**	**K-rep2**	**Con-sensus**	**K-rep1**	**K-rep2**	**Con-sensus**	**K-rep1**	**K-rep2**	**Con-sensus**
canonical/G3L1-7	670	548	449	763	650	531	1090	1124	853	1259	1024	883	220	212	165
longloop	347	348	229	574	426	343	745	810	560	916	740	612	120	99	74
bulges	811	766	453	1346	1133	793	1762	1780	1146	2032	1666	1180	264	212	157
two-quartet	315	343	152	516	454	231	781	704	361	855	715	368	83	72	46
subtotal : canonical & non-canonical motifs	***2143***	***2005***	***1283***	***3199***	***2663***	***1898***	***4378***	***4418***	***2920***	***5062***	***4145***	***3043***	***687***	***595***	***442***
potential G-quadruplex & G>=40%	205	314	59	591	446	233	947	917	364	1163	1037	431	78	58	37
potential G-triplex & G>=40%	27	47	3	77	41	20	129	147	27	168	150	34	8	4	3
G>=40%	342	376	17	573	324	34	824	835	56	969	874	106	46	58	6
subtotal : variants of non-canonical motifs	***574***	***737***	***79***	***1241***	***811***	***287***	***1900***	***1899***	***447***	***2300***	***2061***	***571***	***132***	***120***	***46***
Total – all rG4 motifs	***2753***	***2779***	***1364***	***4440***	***3474***	***2185***	***6354***	***6397***	***3370***	***7456***	***6284***	***3623***	***819***	***715***	***488***
unknown	36	37	2	53	44	2	76	80	3	94	78	9	9	8	1
